# Site‐Selective Ligand Functionalization Reverses Hypsochromic Luminescence Shifts in Platinum(II) Complexes of Benzannulated *NCN*‐Coordinating Ligands

**DOI:** 10.1002/chem.202403766

**Published:** 2024-12-16

**Authors:** Robert J. Ortiz, Esteban Garcia‐Torres, Phillipa L. Brothwood, J. A. Gareth Williams, David E. Herbert

**Affiliations:** ^1^ Department of Chemistry and the Manitoba Institute for Materials University of Manitoba 144 Dysart Road Winnipeg Manitoba R3T 2N2 Canada; ^2^ Department of Chemistry Durham University Durham DH1 3LE UK

## Abstract

Ligands containing phenanthridine (benzo[*c*]quinoline) have presented notable exceptions to the conventional logic that increasing ligand benzannulation leads to bathochromic (red) shifts in the absorption and emission of their coordination complexes. The counterintuitive blue shifts have been attributed to the peculiar structure of phenanthridines, whose ground states are dominated by imine‐bridged biphenyl resonance contributors. These serve to isolate the C=N unit electronically from the rest of the ligand framework and allow the C=N moiety to act as a ‘shock‐absorber’, buffering against larger molecular distortions in a molecule's excited state, and reducing the observed pseudo‐Stokes’ shift. Here, we provide experimental evidence for this assertion in the form of a counterfactual that reverses this trend: substitution at the phenanthridine 6‐position (*i. e*., at the C=N sub‐unit) breaks the phenanthridine's tendency to cause hypsochromic luminescence shifts. The synthesis, full characterization, and comparison of 2‐quinolinyl and 6‐phenanthridinyl exemplars is provided, supported by a detailed theoretical treatment.

## Graphical abstract

**Reversing the Trend**: Phenanthridine (benzo[*c*]quinoline) donors defy conventional logic that increasing ligand benzannulation leads to bathochromic (red) shifts in the absorption and emission of their coordination complexes. Here, we provide a counterfactual that reverses this trend: substitution at the phenanthridine 6‐position (*i. e*., at the C=N sub‐unit) breaks the phenanthridine's tendency to cause hypsochromic luminescence shifts.

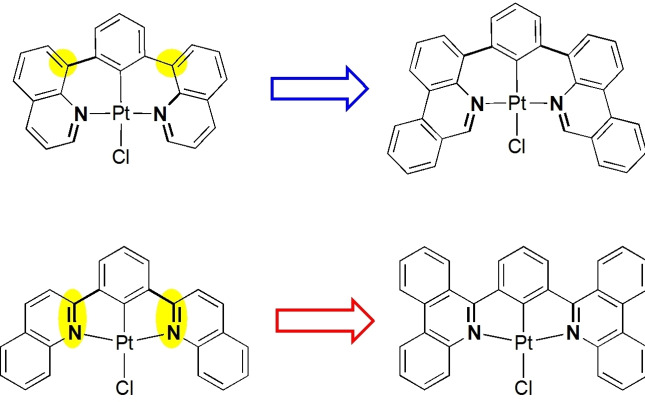

## Introduction

The longstanding interest in phosphorescent chromophores^[1]^ has been driven of late by their numerous useful applications, which include light emitting diodes,[^2^, ^3^, ^4^] chemosensors,[^5^, ^6^] and bioimaging.[^7^, ^8^, ^9^] Pt(II) coordination complexes have played an outsized role in these efforts, thanks to the 5*d* metal's large spin‐orbit coupling (SOC)^[10]^ which facilitates the efficient population of the lowest‐lying triplet excited state (T_1_) via intersystem crossing and its subsequent radiative decay. Combined with a well‐developed understanding of how to tune photophysical properties through ligand design, such species continue to attract significant attention.^[11]^ There remain, however, a number of outstanding challenges. Notably, efficient luminescence of deep red and near‐infrared (NIR) light is an active target both for biological applications and in LED research.^[12]^ A common approach to shifting phosphorescence from mononuclear excited states to longer wavelengths is to stabilize the emissive T_1_ state via ligand π‐extension. Unsaturated ligands including *N*‐heterocycles,^[13]^ cyclometallating aryl rings, and acetylides^[14]^ are common motifs employed to generate emissive Pt(II) coordination complexes, whose luminescent T_1_ states accordingly tend to bear considerable ligand‐involved charge‐transfer character (^3^LLCT or ^3^ILCT), often admixed with metal‐to‐ligand charge transfer (MLCT).^[15]^ Increasing the size of the ligand's π‐system by fusing additional aromatic rings (‘benzannulation’) lowers the energy of such states, increasing the (pseudo) Stokes shift and leading to lower energy phosphorescence.

**Figure 1:**
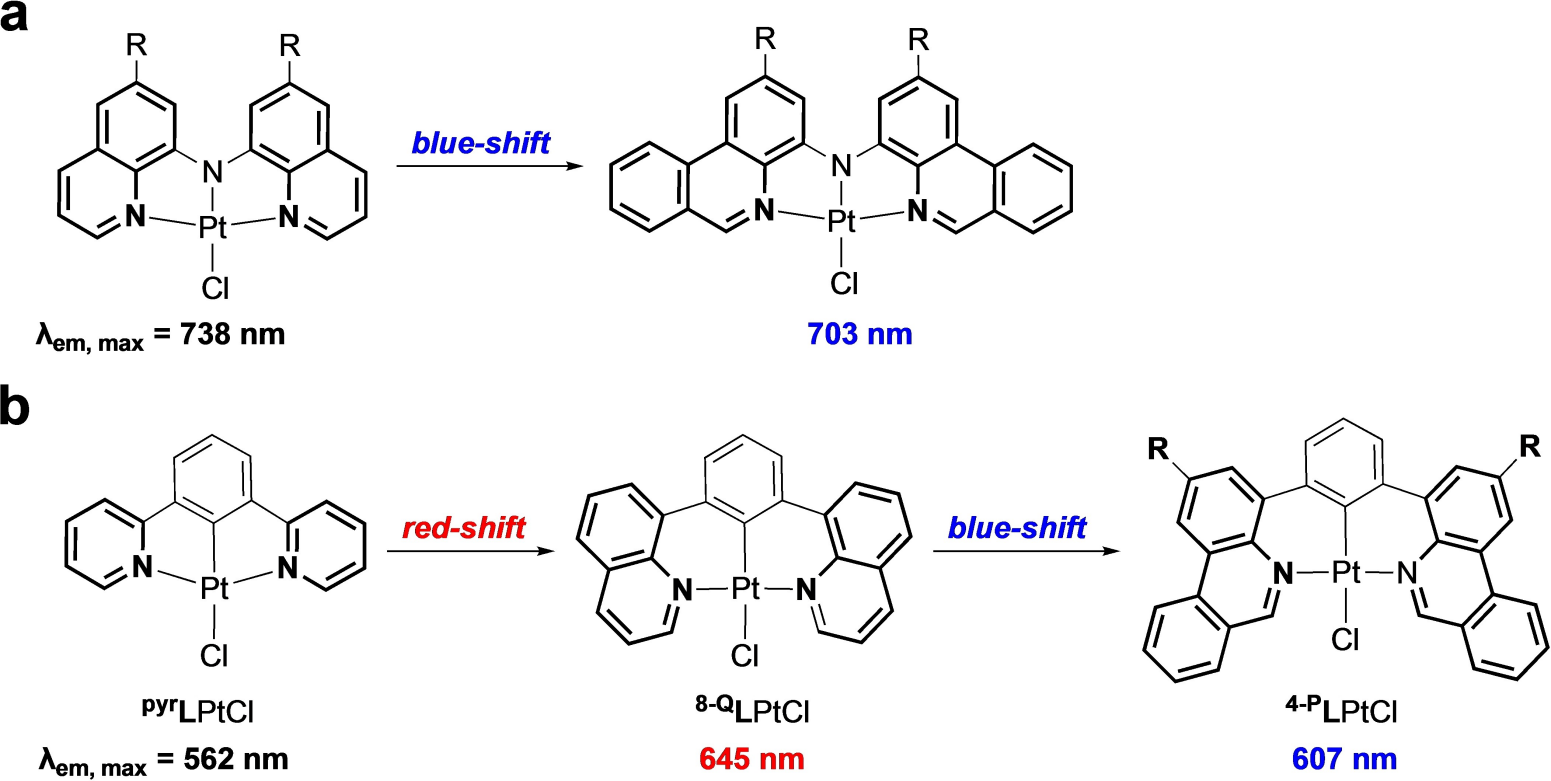
Hypsochromic (‘blue’) shift to emission observed with inclusion of benzannulated phenanthridinyl units in (a) diarylamido scaffolds and (b) cyclometallated analogs of ^**pyr**^ **L**PtCl. In the cyclometallated variants, note that pyridine‐to‐quinoline benzannulation is accompanied by an expected bathochromic (‘red’) shift to the phosphorescence wavelength. In both instances, the phenanthridine donor arms attach to the rest of the ligand framework at the 4‐position.

Research from our group^[16]^ and others^[17]^ has challenged this paradigm. For example, we have shown that site‐selective ligand benzannulation in the form of replacing quinolinyl donors with phenanthridinyl (3,4‐benzoquinoline) units induces a significant shift of emission to *higher* rather than lower energy, decreasing the observed Stokes’ shift.^[18]^ This counterintuitive blue‐shift of the phosphorescence is attributed to heightened molecular rigidity derived from the enhanced ability of the phenanthridinyl units to resist molecular reorganization in their excited states. This effect also enhances the emission efficiency and extends the lifetime of the emissive state by reducing the rate of non‐radiative decay (*k*_nr_). Pt(II) complexes of chelating, phenanthridinyl‐containing, pincer‐like diarylamido scaffolds thus emit in the deep red, with unusually narrow spectral profiles (Figure 1a).^[19]^ A similar effect was observed with phenanthridine‐containing analogs of cyclometallated Pt(II) emitters derived from the highly emissive complex ^**pyr**^ **L**PtCl {^**pyr**^ **L**H=1,3‐di(2‐pyridyl)benzene; Figure 1b}.[^20^, ^21^] While the benzannulated analog ^**8‐Q**^ **L**PtCl {^**8‐Q**^ **L**H=1,3‐di(8‐quinolinyl)benzene} is only weakly emissive ($\lambda_{em}^{max}$ =645 nm; photoluminescence quantum yield Φ=1.6 %) due to sizeable non‐radiative relaxation of its excited state (Σ*k*_nr_=7.0 × 10^4^ s^−1^),^[22]^ the further benzannulated congener ^**4‐P**^ **L**PtCl {^**4‐P**^ **L**H=1,3‐di(4‐phenanthridinyl)benzene} is much more strongly luminescent (Φ=9 %), again emitting higher energy light ($\lambda_{em}^{max}$ =607 nm).^[23]^ In these instances, computational analysis indicated that the particular site‐selective benzannulation in the phenanthridinyl donor arms allows the phenanthridine C=N sub‐unit to absorb the major distortions which the chromophores undergo in their excited states.^[16]^ This buffers the rest of the rings from bigger changes. Thus, while the formation of triplet states in arenes is generally accompanied by considerable distortion,^[24]^ in these phenanthridine‐ligated Pt(II) chromophores, benzannulation destabilizes the triplet excited state by inhibiting what should be an electronically stabilizing distortion. A similar effect has been seen in phosphorescent Cu(I) dimers.^[25]^

Given the aforementioned continued interest in phosphorescent platinum complexes exhibiting efficient emission in the red to NIR,^[26]^ we were curious to see if this counterintuitive hypsochromic shift of emission in phenanthridinyl‐ligated coordination complexes could be manipulated based on where the chelating ligand framework attaches to the benzannulated donor arms. Specifically, we hypothesized that changing the position of functionalization from the 4‐position (*i. e*., on one of the two fused C_6_ rings) to the 6‐position (at the C=N sub‐unit) might impact the photophysical properties (Figure 1).^[22]^ We describe herein two new cyclometallating pincer‐type ligands based on benzannulated quinoline and phenanthridine donors flanking a central phenyl ring. We find that, indeed, in contrast to prior results, shifting the site of functionalization reverses the blue‐shift previously observed comparing quinoline vs. phenanthridine‐supported Pt(II) chromophores,[^18^, ^19^, ^23^] enabling very deep red emission with quite high luminescence quantum yields.

## Results and Discussion

**Scheme 1:**
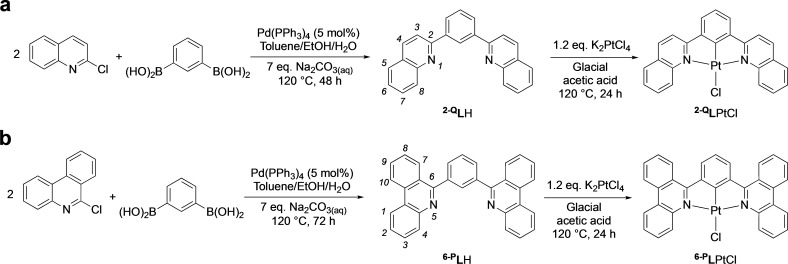
Synthesis of the proligands and cyclometallated complexes. The IUPAC numbering schemes for (a) quinolines and (b) phenanthridines are noted for the proligands in italics.

The pseudo‐Stokes’ shift exhibited by phosphorescent complexes indicates the extent of geometric reorganization that accompanies electronic excitation and the subsequent relaxation to an emissive triplet excited state.^1^, ^[27]^ For the systems noted in Figure 1 wherein we observed the aforementioned counterintuitive blue‐shifted emission, computational analysis revealed that the largest changes to bond lengths in the ligand upon photoexcitation were localized to the C=N sub‐unit in the 4‐phenanthridinyl‐ligated systems;^[18]^ this contrasts with the quinoline analogs wherein the distortions in the excited state proved more evenly spread about the heterocyclic ligand arms. We trace this to the dominant imine‐localized resonance structure of phenanthridines in general,^[16]^ which, when drawn as an ‘imine‐bridged biphenyl’, maximizes the number of aromatic sextets in the molecule.^[28]^ The C=N sub‐unit can be thought of as a sort of ‘shock‐absorber’, elevating the energy of the emissive T_1_ excited state by minimizing excited‐state distortions and at the same time reducing the reorganization energy that accompanies relaxation back to the ground state. To obviate this effect and thus access deeper red‐emitting phosphors, we therefore prepared proligands wherein the phenanthridinyl donors connect to the central cyclometallating ring directly at the C=N sub‐unit (*i. e*., at the 6‐position; Scheme 1).

The two proligands were prepared by Pd‐catalyzed cross‐coupling of either 2‐chloroquinoline or 6‐chlorophenanthridine with benzene‐1,3‐diboronic acid using virtually identical conditions. A slightly longer reaction time helped push conversion higher with the larger ligand. Both proligands were isolated in respectable yields (^**2‐Q**^ **L**H: 61 %; ^**6‐P**^ **L**H: 59 %). The metalated complexes were synthesized by heating a mixture of the corresponding proligand with K_2_PtCl_4_ in acetic acid under an argon atmosphere. Both ^**2‐Q**^ **L**PtCl and ^**6‐P**^ **L**PtCl precipitated from the reaction mixture and were collected by vacuum filtration as light orange powders in 36 % and 47 % yield, respectively. We attribute the relatively forcing conditions required for appreciable conversion to the steric demand of the ^**2‐Q**^ **L** and ^**6‐P**^ **L** ligands which both contain benzannulation *ortho* to the coordinating nitrogens; the CH in the 8‐ and 4‐positions, respectively, are in a position for steric clash with the chloride (*vide infra*). Compared with ^**8‐Q**^ **L** and ^**4‐P**^ **L**, the coordination environment induced by ^**2‐Q**^ **L** and ^**6‐P**^ **L** also contains smaller 5‐membered chelate rings which can impact the extent of ligand‐metal orbital overlap.^[22]^ The solubility of ^**2‐Q**^ **L**PtCl was slightly superior to that of the highly insoluble ^**6‐P**^ **L**PtCl. ^1^H NMR spectroscopy was used to confirm coordination of the ligand through the disappearance of the central aryl proton resonance, which appears as a triplet at 8.97 ppm in ^**2‐Q**^ **L**H, and the appearance of a downfield doublet at 10.01 ppm (*J*_HH_=8.9 Hz). This signal, which integrates to two H nuclei, is assigned to the two protons in the 8‐positions which experience significant deshielding attributed to intramolecular hydrogen‐bonding with the platinum‐ligated chloride {d(Cl−H) ~2.5 Å in both structures; *vide infra*}.

**Figure 2:**
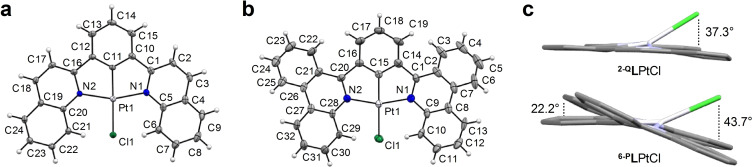
Solid‐state structures of (a) ^**2‐Q**^ **L**PtCl and (b) ^**6‐P**^ **L**PtCl with thermal ellipsoids shown at 50 % and 30 % probability levels, respectively. (c) Alternative “side‐on” views of the two crystal structures highlighting the out‐of‐plane distortion of the chloride ligands and the dihedral angle between the phenanthridinyl moieties and the central phenylenyl ring in ^**6‐P**^ **L**PtCl.

Single crystals suitable for X‐ray diffraction could be obtained for both complexes (Figure 2a–b): ^**2‐Q**^ **L**PtCl by slow evaporation of a dichloromethane solution at −15 °C, and ^**6‐P**^ **L**PtCl by liquid‐liquid diffusion of dichloromethane/toluene mixture at room temperature. The structures confirm the four‐coordinate, distorted square planar geometry expected of Pt(II). The τ_δ_ values calculated for the Pt center in both complexes ^**2‐Q**^ **L**PtCl (τ_δ_=0.306) and ^**6‐P**^ **L**PtCl (τ_δ_=0.313) are far from the ideal value of zero for a square‐planar geometry. This arises from the significant out‐of‐plane distortion of the chlorines −37.3° and 43.7°, respectively, relative to the planes defined by atoms N(1)−N(2)−C(5)−C(20) and N(1)−N(2)−Pt−Cl for ^**2‐Q**^ **L**PtCl and N(1)−N(2)−C(9)−C(28) and N(1)−N(2)−Pt−Cl for ^**6‐P**^ **L**PtCl – evidence of the steric hindrance imposed by the *ortho*‐hydrogens of the *N*‐heterocyclic donors (Figure 2c). Interestingly, aside from the chloride, the quinolinyl analogue ^**2‐Q**^ **L**PtCl is almost completely flat with an angle between the quinolinyl planes and the central aryl ring of just 3.4°. The corresponding bending in ^**6‐P**^ **L**PtCl is more pronounced (22.2°; Figure 2c) but still much less drastic than that observed in ^**8‐Q**^ **L**PtCl^[22]^ or ^**4‐P**^ **L**PtCl and their derivatives (~38–44°).^[23]^ The Pt−C_Ar_ distances are also shorter {^**2‐Q**^ **L**PtCl: 1.915(4) Å; ^**6‐P**^ **L**PtCl 1.876(9) Å; Table 1}. Both complexes show evidence of strong intermolecular π‐stacking interactions in their extended packing (Figures S1, S2), which aligns with the experimental observation of poor solubility. We return to these two observations later, *vis‐à‐vis* the photophysics.

**Table 1:** Selected solid‐state parameters.

Complex	Pt−N_1_/Å	Pt−N_2_/Å	Pt−C_Ar_/Å	Pt−Cl/Å	N_1_−Pt−N_2_ /°	C_Ar_−Pt−Cl /°	C_Ar_−Pt‐ N_1_ /°	C_Ar_−Pt−N_2_ /°	τ_δ_^*c*^
^2‐Q^LPtCl	2.092(3)	2.070(3)	1.915(4)	2.4383(11)	159.02(12)	157.82(11)	79.91(14)	79.99(14)	0.304
^6‐P^LPtCl	2.054(8)	2.056(8)	1.876(9)	2.448(3)	158.8(3)	157.0(3)	80.4(4)	79.8(4)	0.310
^Me,Me‐8‐Q^LPtCl^[a]^	2.035(3)	2.035(3)	1.992(5)	2.451(1)	177.8(2)	180.0	91.1(1)	91.1(1)	0.015
^4‐P^LPtCl^[b]^	2.018(10)	2.019(10)	1.979(13)	2.403(4)	179.3(4)	178.4(4)	90.2(5)	90.3(5)	0.016

^[a] **Me,Me‐8‐Q**^ **L**H=4,6‐dimethyl‐1,3‐di(8‐quinolinyl)benzene. Data from ref. [29]. ^[b]^ Data from ref. [30]. ^[c]^ τ_δ_=$\frac{360-(\alpha +\beta )}{141}$ δ, where δ=β/α following ref. [31].

**Figure 3:**
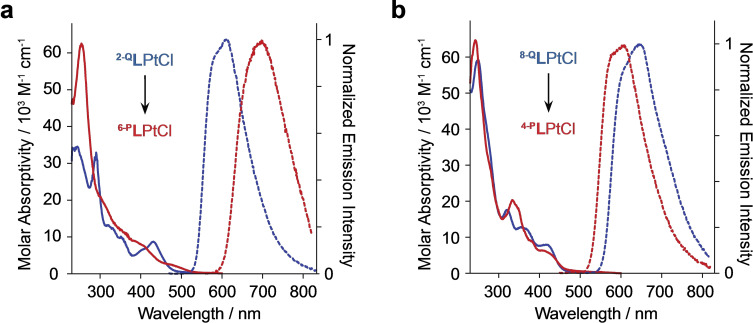
UV‐vis absorption (solid lines) and emission (dashed lines) spectra of (a) ^**2‐Q**^ **L**PtCl and ^**6‐P**^ **L**PtCl highlighting the red shift of absorption/emission with benzannulation (represented by the black arrow) observed for these complexes compared to (b) the nearly isoenergetic absorption and blue shifted emission seen for ^**8‐Q**^ **L**PtCl/^**4‐P**^ **L**PtCl.

Site‐selective π‐extension from quinoline to phenanthridine can have a varied impact on electronic absorption in coordination complexes. The lowest energy absorption can be isoenergetic (as in Pt(II) amido complexes),[^18^, ^19^] or undergo a more conventional shift to lower energy (as in Cu(I) halide dimers)^[25]^ or a counterintuitive blue‐shift to higher energy (as in pseudo‐octahedral Fe(II) complexes of P^N ligands).^[32]^ Here, the lowest energy absorption shifts more significantly to lower energy than in prior examples (${\Delta \lambda }_{abs}^{max}$ =28 nm; ^**2‐Q**^ **L**PtCl 432 nm, ^**6‐P**^ **L**PtCl: 460 nm; Figure 3). This effect is reproduced by density functional theory (DFT) and time‐dependent DFT (TDDFT) modelling which assigns metal‐to‐ligand charge‐transfer (MLCT) character to transitions giving rise to these peaks. We discuss this more below. Comparing the lowest energy MLCT absorption bands of some *N^C^−^^N*‐ligated complexes bearing five‐membered chelate rings, the expected trend of a bathochromic shift accompanying increasing ligand conjugation is observed: λ_MLCT_^**pyr**^ **L**PtCl: 401 nm,^[20]^ ^**2‐Q**^ **L**PtCl: 432 nm, ^**6‐P**^ **L**PtCl: 474 nm (Table 2). Furthermore, when comparing the absorptive properties with their 6‐membered chelate counterparts, both are also red‐shifted: λ_MLCT_^**8‐Q**^ **L**PtCl: 420 nm,^[22]^ ^**4‐P**^ **L**PtCl: 408 nm.^[23]^ Thus, adjusting the site of attachment of the phenanthridine donor has a large effect on ground‐state absorption: while ^**8‐Q**^ **L**PtCl and ^**4‐P**^ **L**PtCl absorb the same low energy light, ^**2‐Q**^ **L**PtCl/^**6‐P**^ **L**PtCl now obey the expected trend of bathochromic shifts in absorption with benzannulation.

**Table 2:** Absorption and Emission Data of Selected Pt(II) Complexes.^[a]^

Complex	Absorption λ_max_/nm (ϵ/10^3^ M^−1^ cm^−1^)	Emission λ_max_ /nm	Φ/10^−2[b]^	τ/μs ^*c*^	*k*_r_/ 10^3^ s^−1[d]^	∑*k*_nr_/10^3^ s^−1[d]^	*k*_Q_^O2^/ 10^8^ M^−1^ s^−1[e]^	Emission^[f]^ 77 K	
								λ_max_/nm	τ/μs
^2‐Q^LPtCl	244 (34.6), 289 (31.3), 321 (13.2), 351 (10.1), 432 (8.7)	580, 610	0.23	5.8 [0.64]	40	130	6.3	546, 585 680^[k]^	13
^6‐P^LPtCl	255 (62.2), 397sh (8.2), 474sh (2.5)	700	0.021	3.7 [1.0]	5.7	270	3.3	585, 648, 692	11
^8‐Q^LPtCl^[g]^	320 (17.5), 356 (12.8), 420 (7.88)	611 (sh), 645	0.016	14 [*h*]	1	70	*h*	575, 626, 676	28
^4‐P^LPtCl^[i]^	243 (73.5), 333 (21.6), 380 (9.12), 408 (6.25)	575 (sh), 607	0.09	16 [1]	6	60	5.0	573, 613	20
^pyr^LPtCl^[j]^	332 (6.51), 380 (8.69), 401 (7.01), 454 (0.27), 485 (0.24)	491, 524, 562	0.60	7.2 [0.5]	83	56	9.1	492, 525, 562	7.0

^[a]^ In degassed CH_2_Cl_2_ at 295 K, except where indicated otherwise. ^[b]^ Photoluminescence quantum yield measured in deoxygenated solution using [Ru(bpy)_3_]Cl_2(aq)_ as the standard; estimated uncertainty in Φ is ±20 %. ^[c]^ Luminescence lifetimes in deoxygenated solution; corresponding values in air‐equilibrated solution are given in square parentheses; estimated uncertainty in τ is ±10 %. ^[d]^ Radiative *k_r_* and nonradiative ∑*k_nr_* rate constants estimated from the quantum yield and lifetime, assuming unit population of the emissive state upon light absorption, through the relationships *k_r_* =Φ/*τ*; *k_nr_* =(1–Φ)/*τ*. ^[e]^ Bimolecular Stern–Volmer constant for quenching by molecular oxygen, estimated from the lifetimes in deoxygenated and air‐equilibrated solution and assuming [O_2_]=2.2 mmol dm^−3^ in CH_2_Cl_2_ at atmospheric pressure of air. ^[f]^ In diethyl ether/isopentane/ethanol (2 : 2 : 1 v/v). ^[g]^ Data for ^**8‐Q**^ **L**PtCl from ref. [22], with revised extinction coefficients. ^[h]^ Emission too weak for the luminescence lifetime of this complex to be measured under air‐equilibrated conditions. ^[i]^ Data for ^**4‐P**^ **L**PtCl (R=CF_3_) are from ref. [23]. ^[j]^ Data for ^**pyr**^ **L**PtCl from ref. [20]. ^[k]^ The band at 680 nm is likely due to aggregation owing to the poor solubility at 77 K. Its relative intensity decreases upon dilution (see Figure S3a), consistent with this interpretation.

Both ^**2‐Q**^ **L**PtCl and ^**6‐P**^ **L**PtCl are brightly luminescent in degassed solution at room temperature, with broad, unstructured emission in the visible region. Comparing ^**2‐Q**^ **L**PtCl and ^**6‐P**^ **L**PtCl, a red shift to the emission maximum is again observed with $\lambda_{em}^{max}$ ~610 nm for ^**2‐Q**^ **L**PtCl (with the 0,0 component partially resolved as a shoulder at 580 nm), compared to $\lambda_{em}^{max}$ =700 nm for ^**6‐P**^ **L**PtCl. When cooled to 77 K the vibrational progression becomes well resolved showing a clear 0,0 band at higher energies for ^**6‐P**^ **L**PtCl. With the more planar ^**2‐Q**^ **L**PtCl (*vide supra*), an aggregate forms at 77 K and the resulting red‐shifted emission due to aggregation dominates the spectrum at ~680 nm (Figure S3a). The self‐quenching for ^**2‐Q**^ **L**PtCl in solution is quite pronounced, with the bimolecular quenching constant determined to be 3.1×10^9^ M^−1^ s^−1^ at 295 K (Figure S5). In contrast, ^**6‐P**^ **L**PtCl, as for ^**4‐P**^ **L**PtCl and indeed ^**8‐Q**^ **L**PtCl, showed no such self‐quenching over the accessible concentration range (Figure S3b), likely due to the more twisted structure of the complex which makes face‐to‐face interactions less favourable. Similar to the absorption, when complexes containing phenanthridine‐based ligands are functionalized at the 4‐position, a blue shift in the emission has typically been observed when compared to their quinoline counterparts.[^18^, ^19^, ^23^] Here, a nice trend of red‐shifted emission comparing pyridine to quinoline to phenanthridine can be observed (^**pyr**^ **L**PtCl=491 nm;^[20]^ ^**2‐Q**^ **L**PtCl=610 nm; ^**6‐P**^ **L**PtCl=700 nm), with a ~90 nm bathochromic shift accompanying each successive benzannulation. For both ^**2‐Q**^ **L**PtCl and ^**6‐P**^ **L**PtCl, the excitation spectra closely match the absorption spectra (Figure S4) indicating that the emitting state is formed approaching unit efficiency.

The quantum yields (^**2‐Q**^ **L**PtCl: 0.23; ^**6‐P**^ **L**PtCl: 0.021) recorded at 295 K in degassed CH_2_Cl_2_ reflect relatively efficient luminescence, with lifetimes (^**2‐Q**^ **L**PtCl: 5.8 μs; ^**6‐P**^ **L**PtCl: 3.7 μs; Table 2) on the order of those seen in related Pt(II) complexes supported by nac‐ac style {*N^N^O*}^−^ ligands also bearing phenanthridinyl donor arms.^[33]^ The microsecond lifetimes and sensitivity of the emission intensity to molecular oxygen in solution are consistent with phosphorescence from a triplet excited state. The yellow emission of ^**2‐Q**^ **L**PtCl is quite intense and in a similar region to (though slightly higher in energy than) that of ^**8‐Q**^ **L**PtCl (λ_em_=645 nm) and ^**4‐P**^ **L**PtCl (λ_em_=607 nm), albeit with a higher quantum yield. The main influence here is the much more efficient radiative decay {*k*_r_: ^**2‐Q**^ **L**PtCl=40000 s^−1^; ^**8‐Q**^ **L**PtCl=1000 s^−1^; ^**4‐P**^ **L**PtCl=6000 s^−1^}, reflecting greater metal character to the emitting state which increases the spin‐orbit coupling and hence *k*_r_.^[34]^ The benzannulated ^**6‐P**^ **L**PtCl shows a sizeable red‐shift to this emission, emitting red‐to‐NIR light at ~700 nm, with a lower quantum yield consistent with the Energy Gap Law.[^35^, ^36^] The greater availability of non‐radiative decay pathways is reflected in the essentially doubled value of the non‐radiative rate constant (∑*k*_nr_: ^**6‐P**^ **L**PtCl: 235000 s^−1^ *cf*. ^**2‐Q**^ **L**PtCl 133000 s^−1^). This higher value of *k*_nr_ may also originate in the more twisted structure of ^**6‐P**^ **L**PtCl (Figure 2). However, also contributing to the lower quantum yield (Φ) is a much smaller *k*_r_ (^**2‐Q**^ **L**PtCl: *k*_r_=40000 s^−1^; ^**6‐P**^ **L**PtCl: *k*_r_=5700 s^−1^). This is consistent with the observation that the filled orbitals of more conjugated ligands do not mix as well with metal orbitals, in turn lowering the extent of metal participation in the emitting state.[^37^, ^38^, ^39^] For a phosphor emitting so deep into the red, the quantum yield observed for ^**6‐P**^ **L**PtCl (Φ=2.1 %) is quite respectable: similar energetically emitting Pt(II) complexes emit with low emission efficiencies.^[40]^

**Figure 4:**
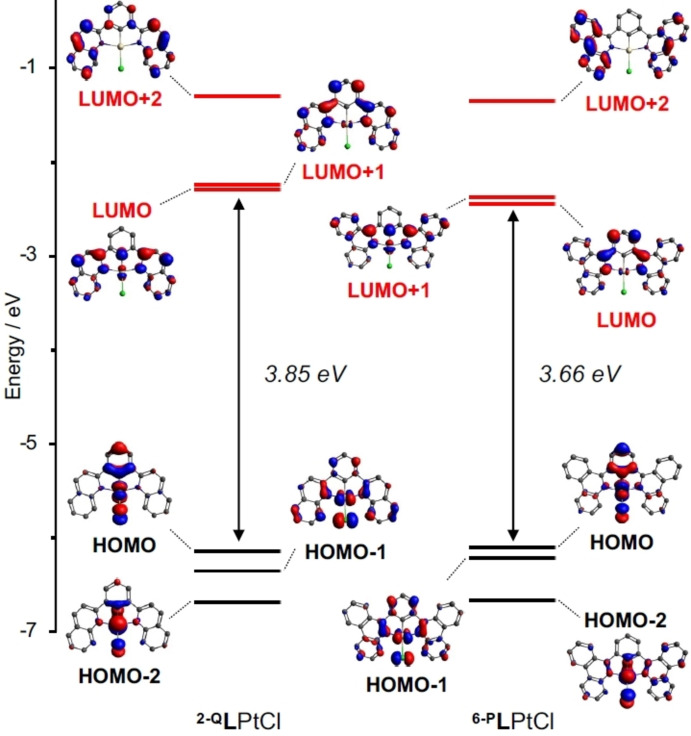
Molecular orbital diagram of selected orbitals and their energies (RIJCOSX‐ZORA‐SMD−M06/def2‐TZVP+SARC/J‐ZORA‐TZVP//SMD−M06 L/def2‐SVP; isosurface=0.05).

DFT calculations of the electronic structures of the optimized ground states of both complexes predicted significant metal character in the highest occupied molecular orbitals (HOMO; ^**2‐Q**^ **L**PtCl=34 %; ^**6‐P**^ **L**PtCl=29 %; Figure 4). The lowest unoccupied molecular orbital (LUMO) is anticipated to lie mostly on the *N*‐heterocyclic moieties. This is consistent with the above‐noted assignment of MLCT character to the lowest energy absorption manifold. There are some differences to the exact character of the LUMO, however. For the smaller quinolinyl π‐system, the LUMO has *b*_1_ symmetry and is symmetric with respect to rotation about the (pseudo) *C*_2_ axis of the molecule which comprises the C−Pt−Cl bonding vector. This orbital additionally presents orbital density at the cyclometallating carbon itself and shows Pt−C π‐anti‐bonding character. The unoccupied MO with corresponding symmetry and character is the LUMO+1 of ^**6‐P**^ **L**PtCl. The LUMO of the phenanthridinyl‐ligated complex still has localized π*(C=N) character on the *N*‐heterocyclic donors, but is anti‐symmetric with respect to rotation about the molecular *C*_2_ axis; *i. e*., the orbital has *a*_2_ symmetry. The LUMO of ^**6‐P**^ **L**PtCl lacks any orbital density at the cyclometallating carbon, instead presenting larger lobes at the two *meta* carbon sites. Such sites have proved of great utility in tuning emission wavelengths and intensity in analogs of ^**pyr**^ **L**PtCl.^[41]^ Focusing on the site of formal benzannulation in ^**2‐Q**^ **L**PtCl that would lead to ^**6‐P**^ **L**PtCl (carbons 3‐ and 4‐ in Scheme 1), the two carbons have local *a*_2_ (π*) symmetry. One can envisage more effective mixing at this site with the *a*_2_ symmetric HOMO of a butadiene fragment, which would raise the energy of the resulting virtual MO (while lowering the energy of the filled combination).^[17]^ At the same time, the mixing between the more energetically stable C=N sub‐unit of phenanthridine and the cyclometallating ring in ^**6‐P**^ **L**PtCl lowers the energy of what was the LUMO+1 MO for ^**2‐Q**^ **L**PtCl, transforming this orbital into the LUMO of ^**6‐P**^ **L**PtCl. Benzannulation is not predicted to impact the HOMOs substantially; most of the impact is on the unoccupied orbitals leading to a narrowing of the calculated HOMO‐LUMO gap of roughly 0.19 eV from ^**2‐Q**^ **L**PtCl **(**3.85 eV) to ^**6‐P**^ **L**PtCl (3.66 eV). The narrower HOMO‐LUMO gap translates to the red‐shift in absorption, again contrasting with prior examples of phenanthridinyl/quinolinyl ligated Pt(II) chromophores where the C=N sub‐unit is left unperturbed.

**Figure 5:**
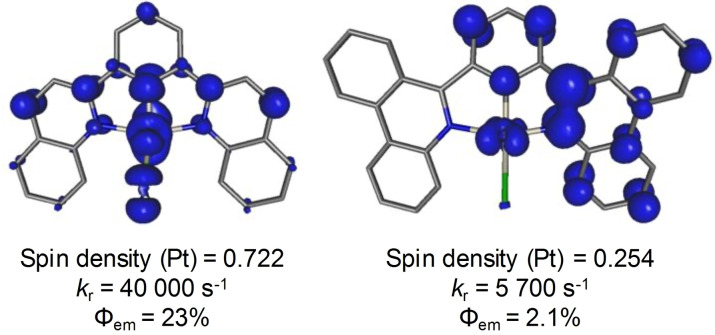
Spin density plots of the optimized emitting T_1_ state of ^**2‐Q**^ **L**PtCl (left) and ^**6‐P**^ **L**PtCl (right) with Mulliken spin population number displaying the spin on the Pt center (RIJCOSX‐ZORA‐SMD−M06/def2‐TZVP+SARC/J‐ZORA‐TZVP//SMD−M06 L/def2‐SVP; isosurface=0.05).

The bathochromic shift to the lowest energy (HOMO→LUMO) absorption is also reproduced by TD‐DFT (Figures S6, S8). These simulations indicate an admixture of both MLCT and intraligand charge‐transfer (ILCT) character to this excitation. In general, the simulated absorption spectra for both complexes accurately reproduce experiment. Electron‐hole density maps (Figures S6, S8) locate the hole mainly on the Pt center, leaking to the cyclometallating phenyl and the chloride, consistent with the structure of the HOMOs (Figure 4). The photoexcited electron is forecasted to localize on the π^*^(C=N) moiety of the *N*‐heterocycle. Following vertical excitation, the complex would undergo intersystem crossing (ISC) to the emissive triplet state. Optimizing the lowest energy triplet excited state supports its ^3^MLCT character and the calculated energy of this emission again closely matches to experiment, reproducing the observed bathochromic shift (Table S7). Interestingly, the calculated spin density maps of the lowest energy triplet state localize the spin at the metal center to a much greater extent in ^**2‐Q**^ **L**PtCl than in ^**6‐P**^ **L**PtCl (Figure 5). In contrast, more of the computed spin density sits on the cyclometallating group and one of the two phenanthridinyl ligand arms in ^**6‐P**^ **L**PtCl, with a large quantity localized on one of the C=N sub‐units. The lowest‐lying triplet state in ^**2‐Q**^ **L**PtCl is much more symmetric and has significantly less spin density on the *N*‐heterocyclic ligand.

This helps explain the much higher *k*_r_ value for ^**2‐Q**^ **L**PtCl: greater Pt involvement increases SOC, making the spin‐forbidden (T_1_→S_0_) transition more probable and thus increasing the radiative rate. This follows the trend where benzannulation alters the energy of the ligand orbitals, lowering the energy of the emissive state but at the same time, reducing metal involvement and decreasing the radiative rate.[^37^, ^38^, ^39^] Comparing the deeper red emission from ^**6‐P**^ **L**PtCl *cf*. ^**2‐Q**^ **L**PtCl with prior examples,[^18^, ^19^, ^23^] it is clear that when the phenanthridine is functionalized at the C=N sub‐unit directly (*i. e*., in the 6‐position), this tricyclic fused‐ring system behaves in the fashion of conventional benzannulation:[^42^, ^43^] both absorption and emission are shifted to lower energy. The counterintuitive hypsochromic effects observed for phenanthridinyl‐ligated Pt(II) phosphors,[^18^, ^19^, ^23^] dimeric Cu(I) halide emitters,^[25]^ and Fe(II) chromophores[^32^, ^44^] require the energetically accessible C=N moiety to be electronically isolated from the rest of the ligand system. This allows it to act as a buffer or ‘shock absorber’ against larger overall molecular distortion in a molecule's excited state.

## Conclusions

Ligand benzannulation is an effective means of controlling photophysical properties of transition metal chromophores, but there has been some debate as to the origin of this effect^[17]^ – and with it, some surprises as to the impact on the energy of absorption/emission based on the site of π‐extension. In this contribution, we were able to reverse the unconventional blue‐shift to the emission energy previously observed in a variety of phenanthridine‐ligated Pt(II) chromophores[^18^, ^19^, ^23^] by altering the site at which the *N*‐heterocyclic unit connects to the rest of the chelating ligand framework. The resulting 6‐substituted 1,3‐(diphenanthridinyl)benzene platinum chloride complex ^**6‐P**^ **L**PtCl both absorbs lower energy light than its 2‐quinolinyl congener ^**2‐Q**^ **L**PtCl, and also emits deeper into the red. This study thus provides direct experimental evidence of the critical role the C=N sub‐unit plays in the triplet excited state and with it, the molecule's emission characteristics. It provides a pathway to continue to access novel deep‐red/NIR phosphorescent materials and to further optimize the efficiency of their luminescence.

## Experimental

### General Information

Air‐sensitive manipulations were carried out either in a N_2_‐filled glove box or using standard Schlenk techniques under argon. 1,3‐Benzene diboronic acid (Combi Blocks), *tetrakis*(triphenyl‐phosphine)palladium (Millipore Sigma), potassium tetrachloridoplatinate (Alfa Aesar), and other common reagents were purchased from commercial suppliers and used without further purification. For moisture‐sensitive manipulations, organic solvents were dried and distilled using appropriate drying agents. 1‐ and 2D NMR spectra were recorded on Bruker Avance 400 MHz or Bruker Avance – III 500 MHz spectrometers. ^1^H, and ^13^C{^1^H} NMR spectra were referenced to residual solvent peaks. High resolution mass spectra (HRMS) were recorded using a Bruker microOTOF‐QIII mass spectrometer.


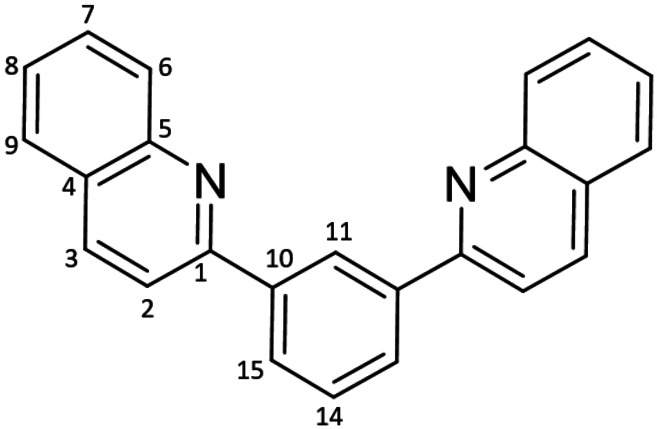


**Synthesis of 1,3‐bis(1‐quinolinyl)benzene** ^**2‐Q**^ **L**H: A thick‐walled, 250 mL Teflon‐stoppered flask was charged with 2‐chloroquinoline (0.500 g, 3.07 mmol), 1,3‐phenylenediboronic acid (0.254 g, 1.53 mmol), sodium carbonate (1.14 g, 10.7 mmol), tetrakis(triphenylphosphine)palladium(0) (0.177 g, 0.153 mmol), H_2_O (20 mL), ethanol (20 mL) and toluene (20 mL). The solution was degassed by sonicating under moderate vacuum for 10 min and then back‐filled with argon. The flask was then sealed and stirred for 48 h in an oil bath set at 120 °C. Then, the solution mixture was reduced in vacuo, diluted in ethyl acetate (250 mL) and washed with brine (x3 250 mL). The solution was then dried over Na_2_SO_4_ and the solvent was removed in vacuo. The resulting solid was stirred in a 1 : 10 H_2_O/MeOH solution for 24 h. The solid was filtered giving a spectroscopically pure beige powder. Yield=0.309 g (61 %) ^1^H NMR (500 MHz, CDCl_3_) δ 8.97 (t, *J*_HH_=1.9 Hz, 1H; *C*_11_‐H), 8.31 (dd, *J*_HH_=7.8, 1.8 Hz, 2H; *C*_15_‐H), 8.28 (d, *J*_HH_=8.7 Hz, 2H; *C*_3_‐H), 8.23 (d, *J*_HH_=8.4 Hz, 2H*; C*_6_‐H), 8.04 (d, *J*_HH_=8.5 Hz, 2H; *C*_2_‐H), 7.87 (d, *J*_HH_=8.1 Hz, 2H; *C*_9_‐H), 7.76 (ddd, *J*_HH_=8.5, 6.9, 1.6 Hz, 2H; *C*_7_‐H), 7.71 (t*, J*_HH_=7.8 Hz, 1H; *C*_14_‐H), 7.56 ppm (ddd, *J*_HH_=8.1, 6.7, 1.2 Hz, 2H; *C*_8_‐H). ^13^C{^1^H} NMR (126 MHz, CDCl_3_) δ 157.1 (*C*_1_), 148.3 (*C*_5_), 140.2 (*C*_10_), 136.9 (*C*_3_), 129.8 (*C*_6_), 129.7 (*C*_7_), 129.4 (*C*_14_), 128.5 (*C*_15_), 127.5 (*C*_9_), 127.3 (*C*_4_), 126.8 (*C*_11_), 126.4 (*C*_8_), 119.2 ppm (*C*_2_).


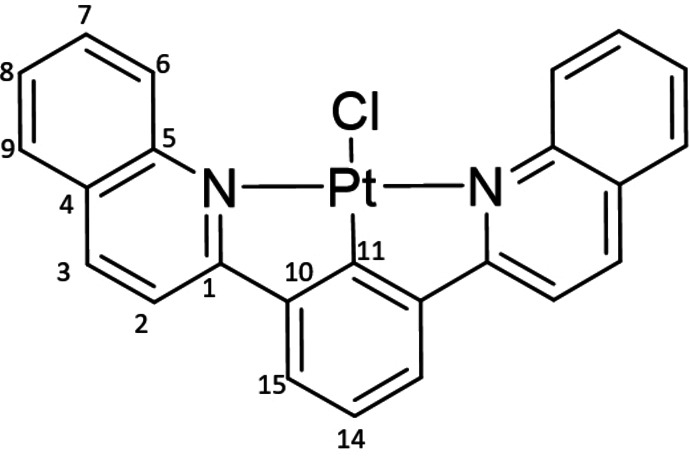


**Synthesis of 1,3‐bis(1‐quinolinyl)benzene platinum chloride** ^**2‐Q**^ **L**PtCl: A 100 mL thick‐walled Teflon‐stoppered flask was charged with ^**2‐Q**^ **L**H (0.050 g, 0.151 mmol), potassium tetrachloroplatinate(II) (0.062 g, 0.151 mmol), and glacial acetic acid (5 mL). The solution was degassed three times using the freeze‐pump‐thaw method and then refilled with argon. The flask was then sealed and stirred for 24 hours in an oil bath set at 120 °C. The solid was collected and washed with H_2_O (~50 mL), acetone (~50 mL), diethyl ether (~50 mL) and CH_2_Cl_2_ (~50 mL), resulting in a spectroscopically pure light orange solid. Yield=0.047 g (36 %) ^1^H NMR (400 MHz, CDCl_3_) δ 9.99 (d, *J*_HH_=8.8 Hz, 2H, *C*_6_‐H), 8.44 (d, *J*_HH_=8.9 Hz, 2H, *C*_3_‐H), 7.93 (t, *J*_HH_=7.3 Hz, 2H, *C*_7_‐H), 7.88 (d, *J*_HH_=8.6 Hz, 2H, *C*_2_‐H), 7.85 (d, *J*_HH_=8.6 Hz, 2H, *C*_15_‐H), 7.70 (d, *J*_HH_=7.9 Hz, 2H, *C*_9_‐H), 7.62 (t, *J*_HH_=7.6 Hz, 2H, *C*_8_‐H), 7.37 ppm (t, *J*_HH_=8.0 Hz, 2H, *C*_14_‐H). UV‐Vis (ϵ) CH_2_Cl_2_, 22 °C: 244 (34600), 289 (31300), 321 (13200), 351 (10100), 432 nm (8670 M^−1^ cm^−1^). HR‐MS (APCI‐TOF) m/z: [M−Cl]^+^ calculated for [C_24_H_15_PtN_2_]^+^: 526.0880; found: 526.0886. Anal. Calc. for C_24_H_15_Cl_1_N_2_Pt_1_(CH_2_Cl_2_)_0.5_: C, 48.69; H, 2.67; N, 4.64 %. Found: C, 48.97; H, 2.63; N, 4.61 %.


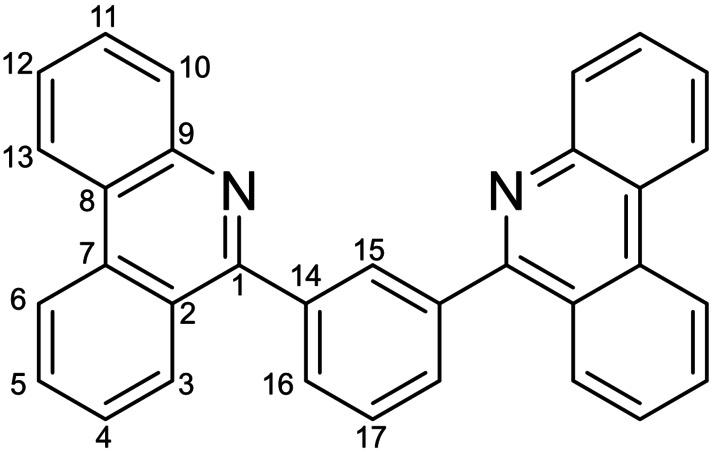


**Synthesis of 1,3‐*bis*(6‐phenanthridinyl)benzene** ^**6‐P**^ **L**H: A 250 mL thick‐walled Teflon‐stoppered flask was charged with 6‐chlorophenanthridine (1.00 g, 4.68 mmol), 1,3‐benzene diboronic acid (0.388 g, 2.34 mmol), toluene (12 mL) and ethanol (23 mL). *Tetrakis*(triphenylphosphine)palladium (0.270 g, 0.234 mmol) was then added, followed by additional toluene (11 mL) and a solution of sodium carbonate (1.74 g, 16.4 mmol) in water (23 mL). The solution was degassed for 10 minutes and then refilled with argon. The flask was then sealed and stirred for 72 h in an oil bath set at 120 °C. The solution was then cooled to room temperature and ethyl acetate (~250 mL) was added. The resulting organic layer was washed with deionized water (3×250 mL), the organic layer was collected, dried over magnesium sulfate, filtered and volatiles were removed *in vacuo*. The residue was taken up in methanol (~20 mL), the mixture was stirred at room temperature for 2 h and then the precipitate collected via vacuum filtration isolating a spectroscopically pure white solid. Yield=0.600 g (59 %). ^1^H NMR (CDCl_3_, 400 MHz, 22 °C): δ 8.68 (dd, *J*_HH_=8.2, 1.1 Hz, 2H; C_13_‐*H*), 8.61 (dd, *J*_HH_=8.3, 1.4 Hz, 2H; C_6_‐*H*), 8.27 (m, 4H; C_3,10_‐*H*), 8.17 (t, *J*_HH_=1.7 Hz, 1H; C_15_‐*H*), 7.96 (dd, *J*_HH_=7.1, 1.7 Hz, 2H; C_16_‐*H*), 7.80 (m, 5H; C_4,12,17_‐*H*), 7.69 (ddd, *J*_HH_=8.3, 7.0, 1.4 Hz, 2H; C_5_‐*H*), 7.62 ppm (ddd, *J*_HH_=8.2, 7.0, 1.2 Hz, 2H; C_11_‐*H*). ^13^C{^1^H} NMR (CDCl_3_, 101 MHz, 22 °C): δ 160.9 (*C*_1_), 143.9 (*C*_7_), 140.0 (*C*_14_), 133.6 (*C*_9_), 131.5 (*C*_15_), 130.7 (*C*_17_), 130.5 (*C*_3_), 130.4 (*C*_16_), 129.1 (*C*_10_), 129.0 (*C*_4_), 128.9 (*C*_12_), 127.4 (*C*_11_), 127.1 (*C*_5_), 125.3 (*C*_8_), 123.9 (*C*_2_), 122.3 (*C*_13_), 122.1 ppm (*C*_6_). HR‐MS (APCI‐TOF) m/z: [M+H]^+^ calculated for [C_34_H_19_F_6_N_2_]^+^: 443.1699; found: 443.1675.

**Synthesis of 1,3‐bis(6‐phenanthridinyl)benzene platinum chloride** ^**6‐P**^ **L**PtCl: A 50 mL Teflon‐stoppered flask was charged with ^**6‐P**^ **L**H (0.025 g, 0.058 mmol), potassium tetrachloridoplatinate (0.024 g, 0.058 mmol), and glacial acetic acid (2 mL). The solution was degassed by 3 freeze‐pump‐thaw cycles. After refilling with argon, the flask was sealed and stirred at reflux in an oil bath set to 120 °C for 24 h. The resulting solution was cooled to room temperature, vacuum filtered, and the precipitate was washed with deionized water (~50 mL), diethyl ether (~50 mL), and acetone (~50 mL) resulting in an orange powder. Yield=0.020 g (47 %). The complex was found to be highly insoluble preventing acquisition of meaningful ^1^H or ^13^C NMR spectra. HR‐MS (APCI‐TOF) m/z: [M−Cl]^+^ calculated for [C_32_H_19_PtN_2_]^+^: 626.1194; found: 626.1184. UV‐Vis (ϵ) CH_2_Cl_2_, 22 °C: 255 (62200), 397sh (8190), 474sh nm (2470 M^−1^ cm^−1^). Anal. Calc. for C_32_H_19_Cl_1_N_2_Pt_1_(H_2_O)_3_(CH_2_Cl_2_)_0.5_: C, 51.46; H, 3.45; N, 3.69 %. Found: C, 51.55; H, 3.66; N, 3.40 %.

## Supporting Information

Additional X‐ray figures, computational discussion, supporting figures and tables; multi‐nuclear NMR and HR‐MS spectra of all new compounds; crystallographic information files containing all X‐ray data. Deposition Number(s) 2389252–2389253 contain(s) the supplementary crystallographic data for this paper. These data are provided free of charge by the joint Cambridge Crystallographic Data Centre and Fachinformationszentrum Karlsruhe Access Structures service.

## Author Contributions

The manuscript was written through contributions of all authors. All authors have given approval to the final version of the manuscript.

## Conflict of Interests

There are no conflicts of interest to declare

1

## Supporting information

As a service to our authors and readers, this journal provides supporting information supplied by the authors. Such materials are peer reviewed and may be re‐organized for online delivery, but are not copy‐edited or typeset. Technical support issues arising from supporting information (other than missing files) should be addressed to the authors.

Supporting Information

Supporting Information

Supporting Information

## Data Availability

The data that support the findings of this study are available in the supplementary material of this article.

## Footnotes

n1 In some instances (*e. g*., ^**pyr**^ **L**PtCl), a weak but well‐defined S_0_→T_1_ absorption band is discernible at high concentration, so that a “true” Stokes’ shift can be quantified. When that is not the case and the lowest absorption band is of singlet character, the observed Stokes’ shift is convoluted with the S_1_−T_1_ energy gap, and we use the term pseudo‐Stokes’ shift to reflect this.
