# Supplementary material for: Site‐Selective Ligand Functionalization Reverses Hypsochromic Luminescence Shifts in Platinum(II) Complexes of Benzannulated NCN‐Coordinating Ligands
Source: Chemistry. 2024 Dec 16;31(6):e202403766. doi: 10.1002/chem.202403766 (PMC11771731; doi:10.1002/chem.202403766)
Supplement: Supplementary file 3 — Supporting Information [file CHEM-31-e202403766-s003.pdf]

Chemistry–A European Journal

Supporting Information

Site-Selective Ligand Functionalization Reverses Hypsochromic Luminescence Shifts in Platinum(II) Complexes of Benzannulated *NCN*-Coordinating Ligands

Robert J. Ortiz, Esteban Garcia-Torres, Phillipa L. Brothwood, J. A. Gareth Williams,* and David E. Herbert*

Supporting Information

for

Site-Selective Ligand Functionalization Reverses Hypsochromic Luminescence Shifts in Platinum(II) Complexes of Benzannulated *NCN*-Coordinating Ligands‡

Robert J. Ortiz,[a] Esteban Garcia-Torres,[a] Phillipa L. Brothwood,[b] J. A. Gareth Williams[b] and David E. Herbert[a]**

[a] Department of Chemistry and the Manitoba Institute for Materials, University of Manitoba, 144 Dysart Road, Winnipeg, Manitoba, R3T 2N2, Canada

*david.herbert@umanitoba.ca

[b] Department of Chemistry, Durham University, Durham, DH1 3LE, UK

*j.a.g.williams@durham.ac.uk

‡ Dedicated to the memory of his Lordship Viceroy Ian Manners

Table of Contents

Experimental Details 4

UV-Vis Absorption and Luminescence Measurements 4

Computational Details 5

X-Ray Crystallography Data 6

6-PLPtCl (CCDC 2389252): 6

2-QLPtCl (CCDC 2389253): 7

Supporting Figures 8

Figure S1. Unit cells from single crystal XRD of (a) $^{2\text{-}Q}$**L**PtCl and (b) $^{6\text{-}P}$**L**PtCl, highlighting the pi-stacking present in the packing. 8

Figure S2. Close up of the π-stacking seen in the crystal packing of (a) $^{2\text{-}Q}$**L**PtCl and (b) $^{6\text{-}P}$**L**PtCl. 8

Figure S3. (a) Emission spectra of $^{2\text{-}Q}$**L**PtCl in EPA at 77 K, excitation wavelength = 454 nm, at decreasing concentrations (black, red, orange, green and blue lines show the spectra for successive 4x-dilutions). The relative intensity of the low-energy band is seen to decrease with decreasing concentration, suggesting that it is due to aggregation. (b) The emission spectrum of $^{6\text{-}P}$**L**PtCl in EPA at 77 K, the form of which shows no concentration dependence. (EPA = diethyl ether / isopentane / ethanol, 2:2:1 v/v). 9

Figure S4. Overlay of the excitation spectra and absorption spectra for (a) $^{2\text{-}Q}$**L**PtCl and (b) $^{6\text{-}P}$**L**PtCl. 9

Figure S5. Plot of the observed rate constant of decay of emission versus concentration for $^{2\text{-}Q}$**L**PtCl in CH_2Cl_2 at 295 K, data points shown as + ... 10

Table S1. Comparison of experimentally and computationally[a] determined bond distances (Å) and angles (°) for $^{2\text{-}Q}$**L**PtCl and $^{6\text{-}P}$**L**PtCl. 10

Figure S6. Comparison of the experimental (-) with the computed TD-DFT spectra (---), and vertical energy transitions (red solid line) for $^{2\text{-}Q}$**L**PtCl in dichloromethane with generated electron-hole density maps showing the character of selected major transitions (RIJCOSX-ZORA-SMD-M06/def2-TZVP+SARC/J-ZORA-TZVP//SMD-M06L/def2-SVP; isosurface = 0.002). Only transitions with oscillator strength > 0.045 are shown. 11

Table S2. TD-DFT calculated electronic transitions along with their corresponding excitation energies and ocillator strengths (f_{osc} > 0.045) for $^{2\text{-}Q}$**L**PtCl (contributions > 10%) 12

Table S3. Fragment contributions (%) to the frontier MOs of $^{2\text{-}Q}$**L**PtCl, using Hirshfeld[15] atomic population method.[a] 12

Figure S7. Expanded selection of molecular orbitals of $^{2\text{-}Q}$**L**PtCl (isosurface = 0.05). 13

Figure S8. Comparison of the experimental (-) with the computed TD-DFT spectra (---), and vertical energy transitions (red solid line) for $^{6\text{-}P}$**L**PtCl in dichloromethane with generated electron-hole density maps showing the character of selected major transitions (RIJCOSX-ZORA-SMD-M06/def2-

TZVP+SARC/J-ZORA-TZVP//SMD-M06L/def2-SVP; isosurface = 0.002). Only transitions with oscillator strength > 0.013 are shown. 14

Table S4. TD-DFT calculated electronic transitions along with their corresponding excitation energies and ocillator strengths (f_{osc} > 0.013) for $^{6\text{-}P}$**L**PtCl (contributions > 10%). 15

Table S5. Fragment contributions (%) to the frontier MOs of $^{6\text{-}P}$**L**PtCl using Hirshfeld[15] atomic population method.[a] 15

Figure S9. Expanded selection of molecular orbitals of $^{6\text{-}P}$**L**PtCl (isosurface = 0.05). 16

Table S6. DFT (SMD-M06L/def2-SVP) optimized ground state and lowest excited triplet state bond lengths (Å) and angles (°). 17

Table S7. Calculated photophysical parameters from single point calculations.[a] 17

Supporting Spectroscopic Figures 18

Figure S10. ^{1}H NMR ($CDCl_3$, 400 MHz, 22°C) of $^{6\text{-}P}$**LH.** 18

Figure S11. ^{13}C{H} NMR ($CDCl_3$, 101 MHz, 22 °C) of $^{6\text{-}P}$**L**H. 19

Figure S12. ^{1}H-^{1}H COSY NMR of $^{6\text{-}P}$**L**H. 20

Figure S13. ^{13}C{H}-^{1}H HSQC NMR of $^{6\text{-}P}$**L**H. 21

Figure S14. ^{13}C{H}-^{1}H HMBC NMR of $^{6\text{-}P}$**L**H. 22

Figure S15. HRMS (APCI-TOF) of $^{6\text{-}P}$**L**H. 23

Figure S16. ^{1}H NMR ($CDCl_3$, 500 MHz, 22°C) of $^{2\text{-}Q}$**LH.** 23

Figure S17. ^{13}C{H} NMR ($CDCl_3$, 126 MHz, 22 °C) of $^{2\text{-}Q}$**L**H**.** 24

Figure S18. ^{1}H-^{1}H COSY NMR of $^{2\text{-}Q}$**L**H. 25

Figure S19. ^{13}C{H}-^{1}H HSQC NMR of $^{2\text{-}Q}$**L**H. 26

Figure S20. ^{13}C{H}-^{1}H HMBC NMR of $^{2\text{-}Q}$**L**H. 27

Figure S21. HRMS (APCI-TOF) of $^{6\text{-}P}$**L**PtCl. 28

Figure S22. ^{1}H NMR ($CDCl_3$, 400 MHz, 22°C) of $^{2\text{-}Q}$**L**PtCl**.** 29

Figure S23. ^{1}H NMR ($CDCl_3$, 400 MHz, 22°C) zoom into aromatic region of $^{2\text{-}Q}$**L**PtCl**.** 30

Figure S24. HRMS (APCI-TOF) of $^{2\text{-}Q}$**L**PtCl. 31

ENERGIES AND REACTION COORDINATES 32

References 41

Experimental Details

UV-Vis Absorption and Luminescence Measurements

Absorption spectra were measured on a Biotek Instruments XS spectrometer, using quartz cuvettes of 1 cm pathlength. Steady-state luminescence spectra were measured using a Jobin Yvon FluoroMax-2 spectrofluorimeter, fitted with a red-sensitive Hamamatsu R928 photomultiplier tube; the spectra shown are corrected for the wavelength dependence of the detector, and the quoted emission maxima refer to the values after correction. Samples for emission measurements were contained within quartz cuvettes of 1 cm pathlength modified with appropriate glassware to allow connection to a high-vacuum line. Degassing was achieved via a minimum of three freeze-pump-thaw cycles whilst connected to the vacuum manifold; final vapor pressure at 77 K was $< 5 \times 10^{-2}$ mbar, as monitored using a Pirani gauge. Measurements were made under this vacuum followed by corresponding aerated measurements upon allowing the solutions to equilibrate with air. Luminescence quantum yields were determined through the following equation:

$$\Phi_{sample} = (I_{sample} / I_{std}) \times (A_{std}/A_{sample}) \times (n_{sample}/n_{std})^2 \times \Phi_{std}$$

where I_{sample} and I_{std} are the integrated areas under the corrected emission spectra of sample and standard, A_{sample} and A_{std} are the respective absorbances at the excitation wavelength employed, n_{sample} and n_{std} are the refractive indices of CH_2Cl_2 and H_2O respectively, and Φ_{std} is the quantum yield of the standard. The standard used was $[Ru(bpy)_3]Cl_2$ (bpy = 2,2'-bipyridine) in air-equilibrated aqueous solution, for which the now widely accepted value of Φ is 0.040±0.002.[1] The estimated uncertainty on the quantum yields obtained in this way on the instrumentation employed is up to ±20%.

The luminescence lifetimes of the complexes < 10 μs were measured by time-correlated single-photon counting, following excitation at 405 nm with a pulsed-diode laser. The emitted light was detected at 90° using a Peltier-cooled R928 PMT after passage through a monochromator. Longer lifetimes were measured following excitation with a microsecond-pulsed xenon lamp and detection using the same PMT

operating in multichannel scaling mode. For all measurements, the decays were much longer than the instrument response, and data were therefore analyzed by tail fitting rather than by deconvolution of the response function. The estimated uncertainty in the quoted lifetimes is ±10% or better. Bimolecular rate constants for quenching by molecular oxygen, k_Q, were determined from the lifetimes in degassed and air-equilibrated solution, taking the concentration of oxygen in CH_2Cl_2 at 0.21 atm O_2 to be 2.2 mmol dm^{-3}.[2]

Computational Details

All computational works was carried out using ORCA version 5.0.2.[3,4] DFT optimizations were performed at the M06L/def2SVP[5–7] level of theory, using the SMD[8] solvent model (CH_2Cl_2). TD-DFT and single point calculations were performed at the M06/def2TZVP[5–7] and with the SMD[8] solvent model with CH_2Cl_2. For all TD-DFT calculations the 'resolution of identity with chain-of-sphere' approximation was used with a special grid on Pt (RIJCOSX; intaccx: 4.34, 4.34, 4.67; gridx: 2,2,2; specialgridintacc: 9),[9] scalar relativistic effects using the two-component, zeroth order regular approximation (ZORA)[10] as implemented in ORCA, along with the SARC-TZVP[7] and corresponding auxiliary basis sets[10–14] on the heavy element Pt. SCF convergence and grid criteria were set to TightSCF and defgrid3, respectively. Molecular orbital analyses were carried out using the Hirshfeld partition method[15] available in Multiwfn software.[16] Avogadro[17] was employed to visualize the molecular orbitals. The results of TD-DFT were analyzed using Multiwfn[16] and spin density maps were generated using Gabedit.[18] To calculate ground-state, excited state, and reorganization energies, a previously established[19,20] protocol was followed. First, the S_0 geometry was first optimized by restricted DFT (charge = 0, multiplicity = 1) using the crystal structure coordinates as starting input. The results were compared to the crystal structure to verify the accuracy of the level of theory. Using this verified method, the rest of the complexes S_0 geometries were optimized and compared to crystal structures where available. The T_1 geometries were optimized with unrestricted DFT (charge = 0, multiplicity = 3) using the optimized S_0 geometry as starting input.

Frequency calculations were carried out with each optimization to confirm that these structures are at a minimum. Second, the electronic energies, $E(S_0)$ and $E(T_1)$, obtained from the single point calculations of S_0 and T_1, in their respective minimum, were used to estimate the adiabatic energy (E^{adia}), where $E^{adia} = E(T_1) - E(S_0)$. (3) TD-DFT was then carried out on the first 50 $S_0 \rightarrow S_n$ singlet-singlet transitions with the restricted formalism with charge = 0, and multiplicity = 1. Population analysis was performed to determine the relative molecular fragment contributions to the frontier MOs which contribute to the electronic transitions. (4) $E^{vert\text{-}phos}$ ($T_1 \rightarrow T_1@S_0$) was estimated as the ΔSCF between single point energies of the T_1 (charge =0, multiplicity = 3) and $T_1@S_0$ (charge = 0, multiplicity = 1) both at the optimized T_1 geometry.

X-Ray Crystallography Data

X-ray crystal structure data was collected from multi-faceted crystals of suitable size and quality selected from a representative sample of crystals of the same habit using an optical microscope. In each case, crystals were mounted on MiTiGen loops and data collection carried out in a cold stream of nitrogen (150 K; Bruker D8 QUEST ECO; Mo Kα radiation). All diffractometer manipulations were carried out using Bruker APEX3 software.[21] Structure solution and refinement was carried out using XS, XT and XL software, embedded within the Olex2 GUI.[22] For each structure, the absence of additional symmetry was confirmed using ADDSYM incorporated in the PLATON program.[23]

6-PLPtCl (CCDC 2389252): X-ray quality crystals were grown from layered diffusion of toluene into a dichloromethane solution of the compound at room temperature. Orange block; $C_{24}H_{15}N_2Pt$ 526.48 g/mol, monoclinic, space group $P2_1/c$; a = 10.500(4) Å, b = 16.156(5) Å, c = 14.869(3) Å, α = 90º, β = 90.614(17), γ = 90°, V = 2522.2(13) Å^3; Z = 4, ρ_{calcd} = 1.743 g cm^{-3}; crystal dimensions 0.1 x 0.1 x 0.05 mm; $2\theta_{max}$ = 26.388°; 23911 reflections, 3242 independent (R_{int} = 0.0554, intrinsic phasing; absorption coefficient (μ = 5.693 mm^{-1}), absorption correction semi-empirical from equivalents (SADABS);

refinement (against F_o^2) with SHELXTL V6.1, 325 parameters, 0 restraints, $R_1 = 0.0554$ ($I > 2\sigma$) and $wR_2 = 0.1177$ (all data), Goof = 1.021, residual electron density 1.238 Å^{-3}.

2-QLPtCl (CCDC 2389253): X-ray quality crystals were grown from layered diffusion of toluene into a dichloromethane solution of the compound at room temperature. Orange needle; $C_{24}H_{15}N_2Pt$ 526.48 g/mol, monoclinic, space group $P2_1/c$; $a = 9.499(2)$ Å, $b = 13.015(3)$ Å, $c = 14.6129(14)$ Å, $\alpha = 90^o$, $\beta = 101.376(12)$, $\gamma = 90°$, V = 1771.1(6) Å^3; Z = 4, $\rho_{calcd} = 2.107$ g cm^{-3}; crystal dimensions 0.17 x 0.024 x 0.020 mm; $2\theta_{max} = 54.972°$; 21226 reflections, 4023 independent ($R_{int} = 0.0212$, intrinsic phasing; absorption coefficient ($\mu = 8.087$ mm^{-1}), absorption correction semi-empirical from equivalents (SADABS); refinement (against F_o^2) with SHELXTL V6.1, 325 parameters, 0 restraints, $R_1 = 0.0183$ ($I > 2\sigma$) and $wR_2 = 0.0419$ (all data), Goof = 1.060, residual electron density 0.542 Å^{-3}.

Supporting Figures

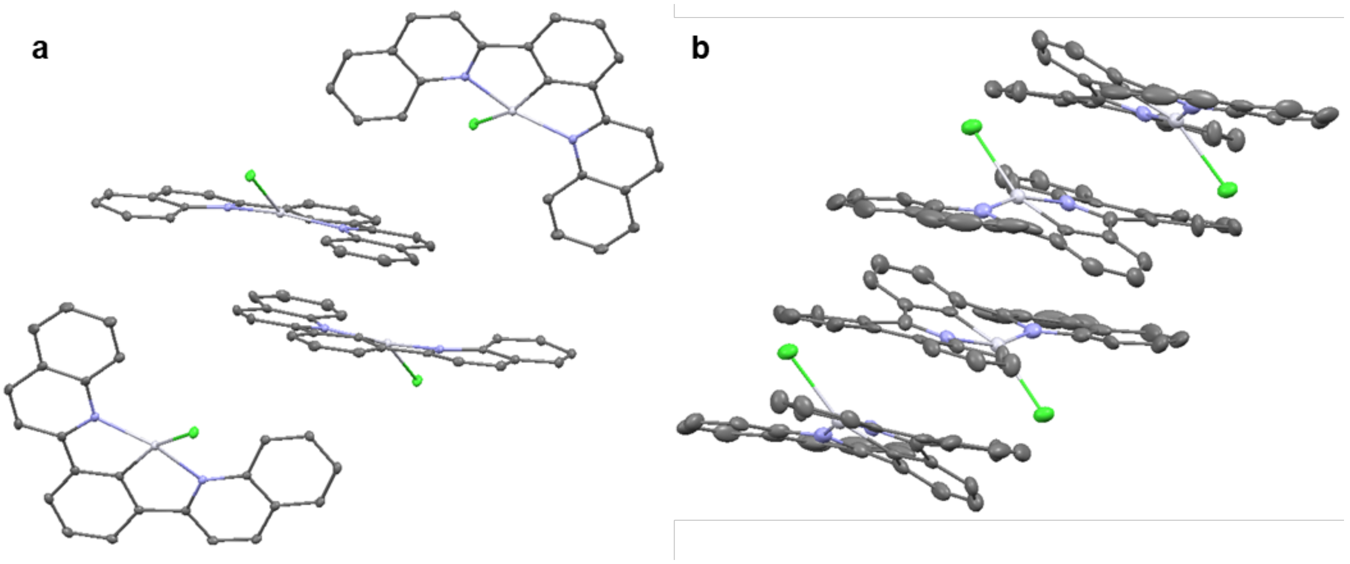

Figure S1. Unit cells from single crystal XRD of (a) $^{2\text{-}Q}$**L**PtCl and (b) $^{6\text{-}P}$**L**PtCl, highlighting the pi-stacking present in the packing.

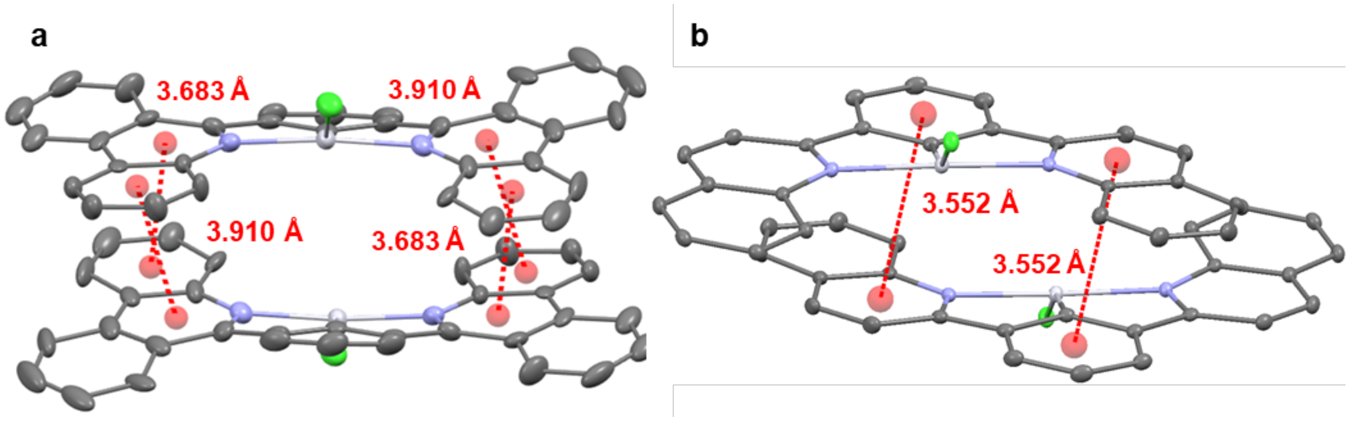

Figure S2. Close up of the π-stacking seen in the crystal packing of (a) $^{2\text{-}Q}$**L**PtCl and (b) $^{6\text{-}P}$**L**PtCl.

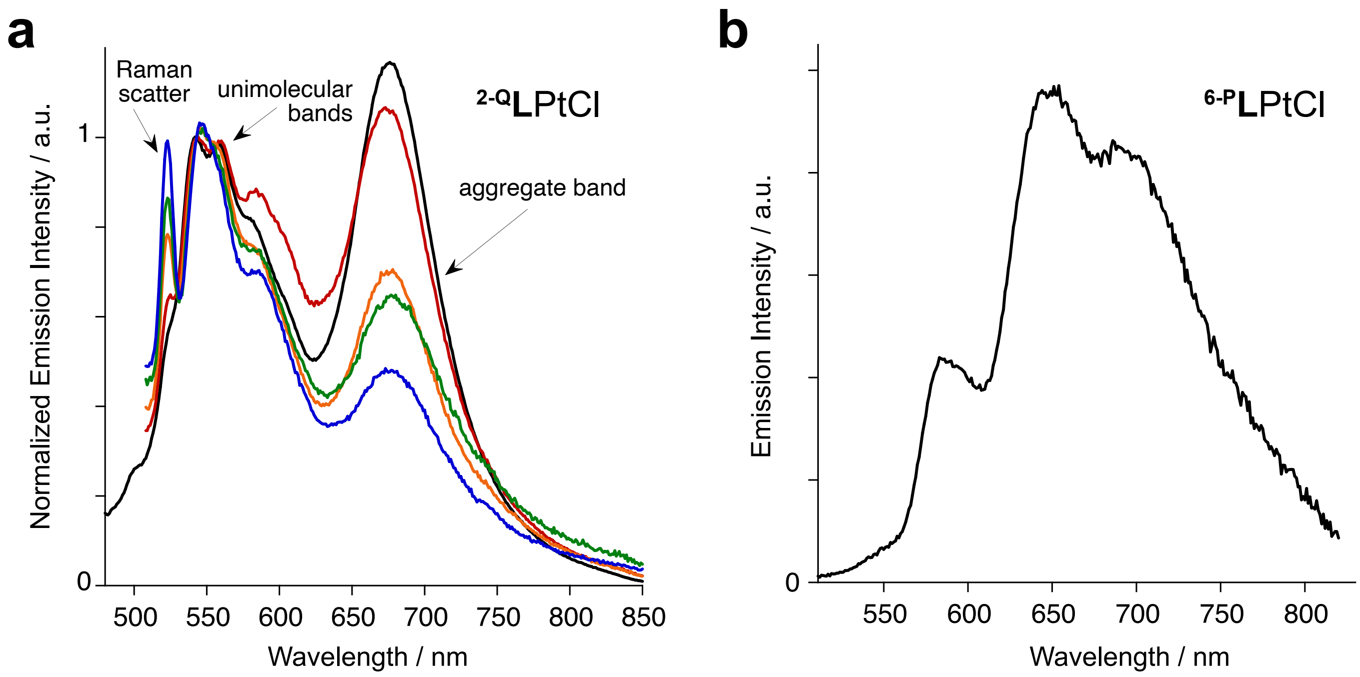

Figure S3. (a) Emission spectra of **$^{2\text{-}Q}$L**PtCl in EPA at 77 K, excitation wavelength = 454 nm, at decreasing concentrations (black, red, orange, green and blue lines show the spectra for successive 4x-dilutions). The relative intensity of the low-energy band is seen to decrease with decreasing concentration, suggesting that it is due to aggregation. (b) The emission spectrum of **$^{6\text{-}P}$L**PtCl in EPA at 77 K, the form of which shows no concentration dependence. (EPA = diethyl ether / isopentane / ethanol, 2:2:1 v/v).

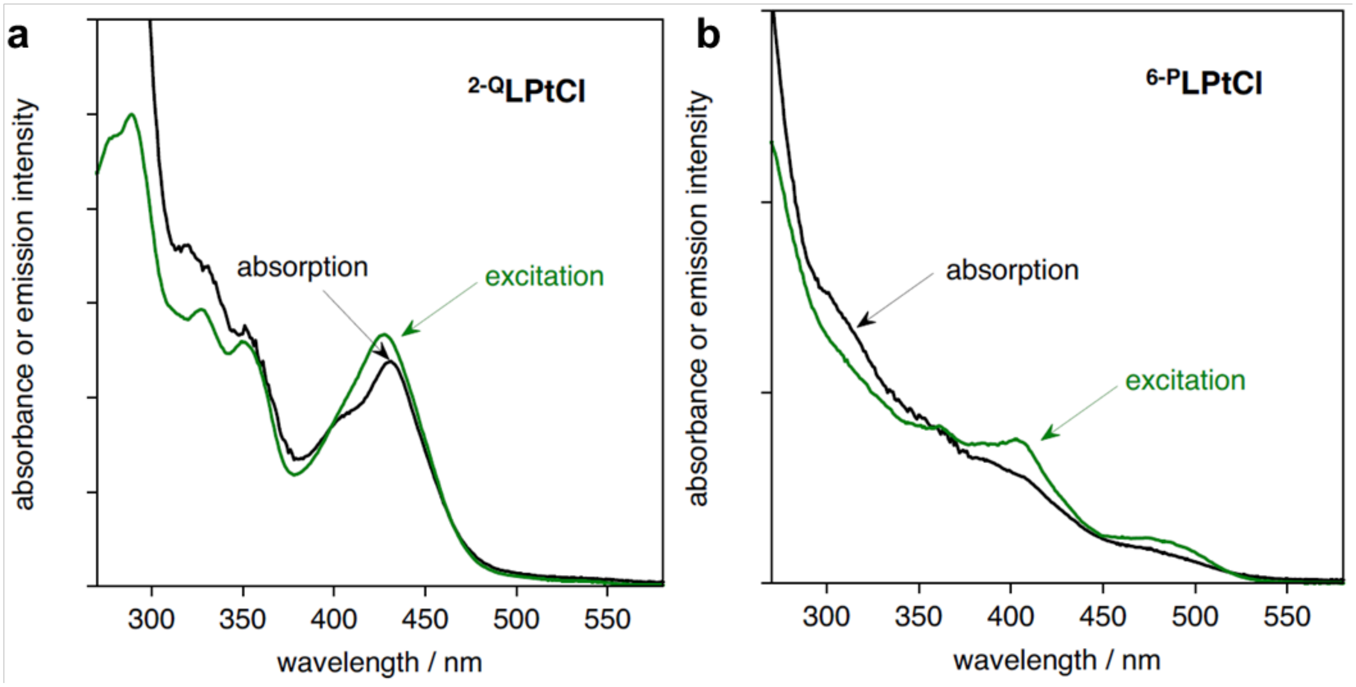

Figure S4. Overlay of the excitation spectra and absorption spectra for (a) **$^{2\text{-}Q}$L**PtCl and (b) **$^{6\text{-}P}$L**PtCl.

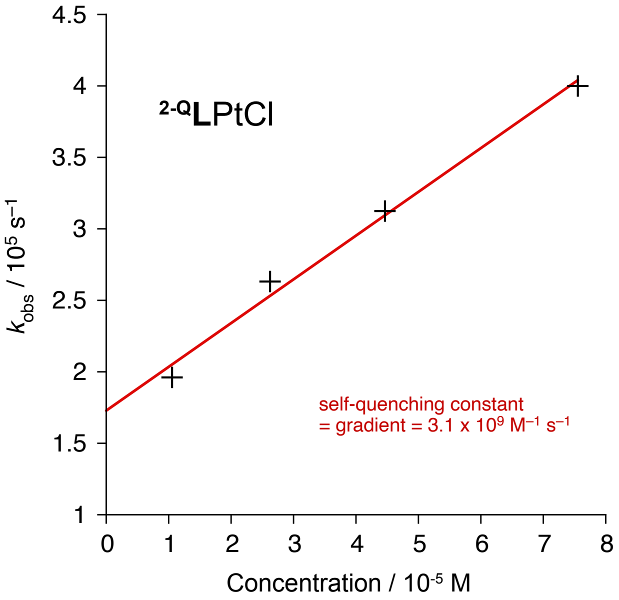

Figure S5. Plot of the observed rate constant of decay of emission versus concentration for $^{2\text{-}Q}$**L**PtCl in CH_2Cl_2 at 295 K, data points shown as +.

Table S1. Comparison of experimentally and computationally[a] determined bond distances (Å) and angles (°) for $^{2\text{-}Q}$**L**PtCl and $^{6\text{-}P}$**L**PtCl.

**Bond/Å**	$^{2\text{-}Q}$**L**PtCl			$^{6\text{-}P}$**L**PtCl		
	**XRD**	**DFT**	**|Δd|**	**XRD**	**DFT**	**|Δd|**
Pt-N_1	2.091	2.123	0.032	2.054	2.103	0.049
Pt-N_2	2.069	2.122	0.053	2.056	2.103	0.047
Pt-C[b]	1.91	1.917	0.007	1.876	1.907	0.031
Pt-Cl	2.438	2.519	0.081	2.448	2.518	0.070
MAD[c]			0.043			0.049
**Angle/°**	**XRD**	**DFT**	**|Δd|**	**XRD**	**DFT**	**|Δd|**
N_1-Pt-N_2	159.0	158.7	0.3	158.8	158.5	0.3
N_1-Pt-Cl	101.8	100.3	1.5	98.2	100.4	2.2
N_2-Pt-C	80.0	79.7	0.3	79.8	79.8	0.0
C-Pt-Cl	157.7	159.0	1.3	157.0	158.8	1.8
MAD[c]			0.8			1.1

[a] (SMD-M06L/def2-SVP)

[b] C_{11} for $^{2\text{-}Q}$**L**PtCl; C_{15} for $^{6\text{-}P}$**L**PtCl

[c] Mean absolute deviation

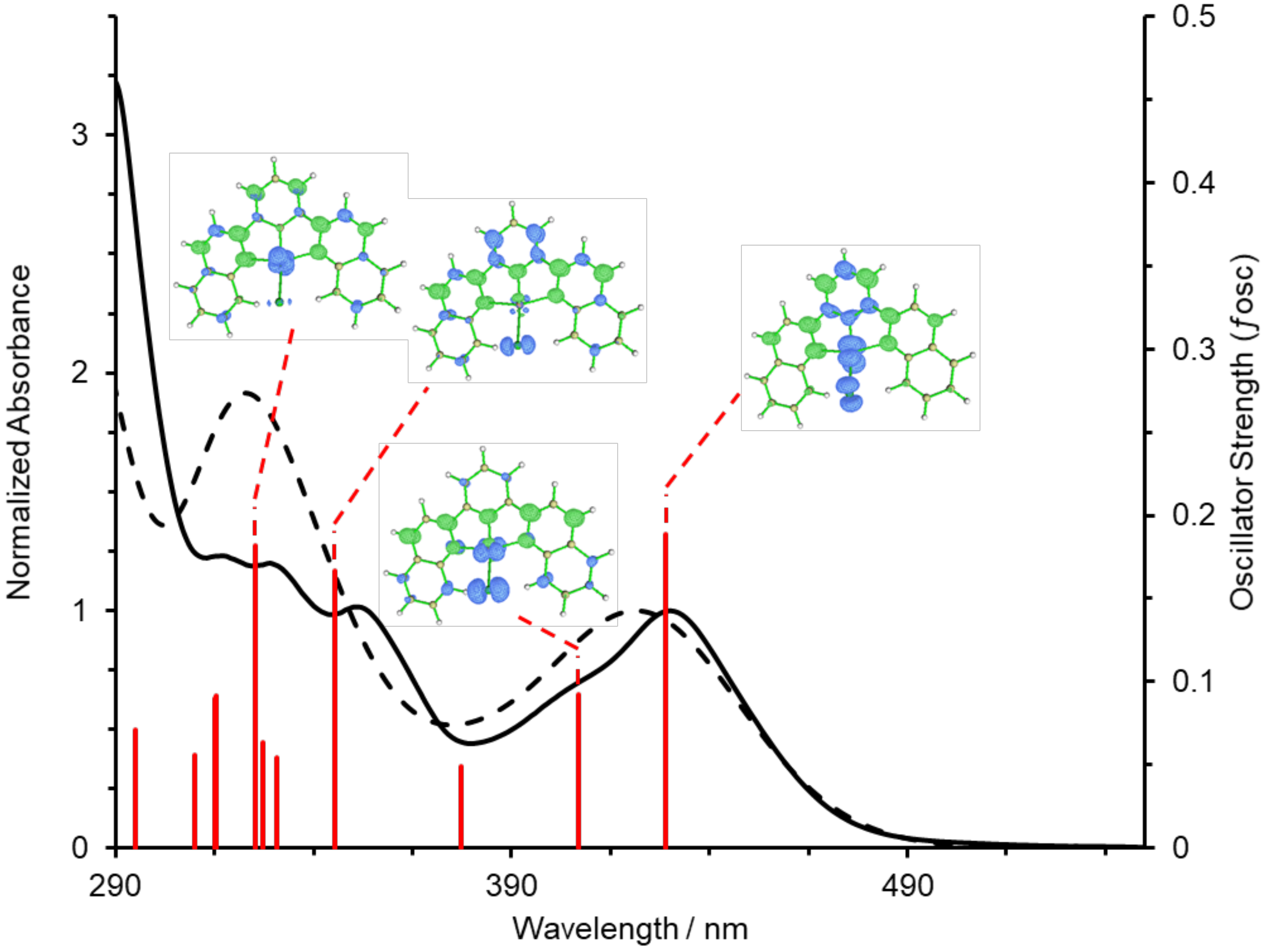

Figure S6. Comparison of the experimental (-) with the computed TD-DFT spectra (---), and vertical energy transitions (red solid line) for **$^{2\text{-}Q}$L**PtCl in dichloromethane with generated electron-hole density maps showing the character of selected major transitions (RIJCOSX-ZORA-SMD-M06/def2-TZVP+SARC/J-ZORA-TZVP//SMD-M06L/def2-SVP; isosurface = 0.002). Only transitions with oscillator strength > 0.045 are shown.

Table S2. TD-DFT calculated electronic transitions along with their corresponding excitation energies and oscillator strengths (f_{osc} > 0.045) for $^{2\text{-}Q}$**L**PtCl (contributions > 10%).

No.	E / nm	f_{osc}	Composition
2	429.0	0.1889	H→L+1 (90%)
3	406.9	0.0922	H-1→L (88%)
5	377.3	0.0494	H-1→L+1 (84%)
7	345.4	0.1667	H-3→L (87%)
8	330.8	0.0545	H-5→L (64%), H-4→L+1 (23%)
9	327.4	0.0631	H-4→L (62%), H-3→L+1 (18%)
10	325.3	0.1816	H-3→L+1 (57%), H-4→L (15%)
11	315.4	0.0916	H-4→L+1 (20%), H→L+5 (16%), H-5→L+1 (11%)
12	315.1	0.0897	H-4→L+1 (23%), H→L+5 (17%), H-5→L+1 (10%)
14	310.0	0.0563	H→L+2 (82%), H-4→L+1 (10%)
16	294.9	0.0708	H-2→L+5 (30%), H-1→L+2 (21%)
20	288.8	0.0866	H-6→L+1 (54%), H-1→L+3 (15%)

Table S3. Fragment contributions (%) to the frontier MOs of $^{2\text{-}Q}$**L**PtCl, using Hirshfeld[15] atomic population method.[a]

MO	E (eV)	Pt	Cl	C=N$_1$	C=N$_2$	Ar(N1)	Ar(N2)	Ar$_{phenyl}$
L+2	-1.30	1	0	3	4	38	38	16
L+1	-2.25	2	0	13	14	19	21	31
L	-2.29	8	1	14	12	27	25	13
H	-6.14	34	15	2	2	7	7	34
H-1	-6.35	22	20	7	7	16	16	12
H-2	-6.69	56	11	3	3	6	6	15

[a]RIJCOSX-ZORA-SMD-M06/def2-TZVP+SARC/J-ZORA-TZVP//SMD-M06L/def2-SVP

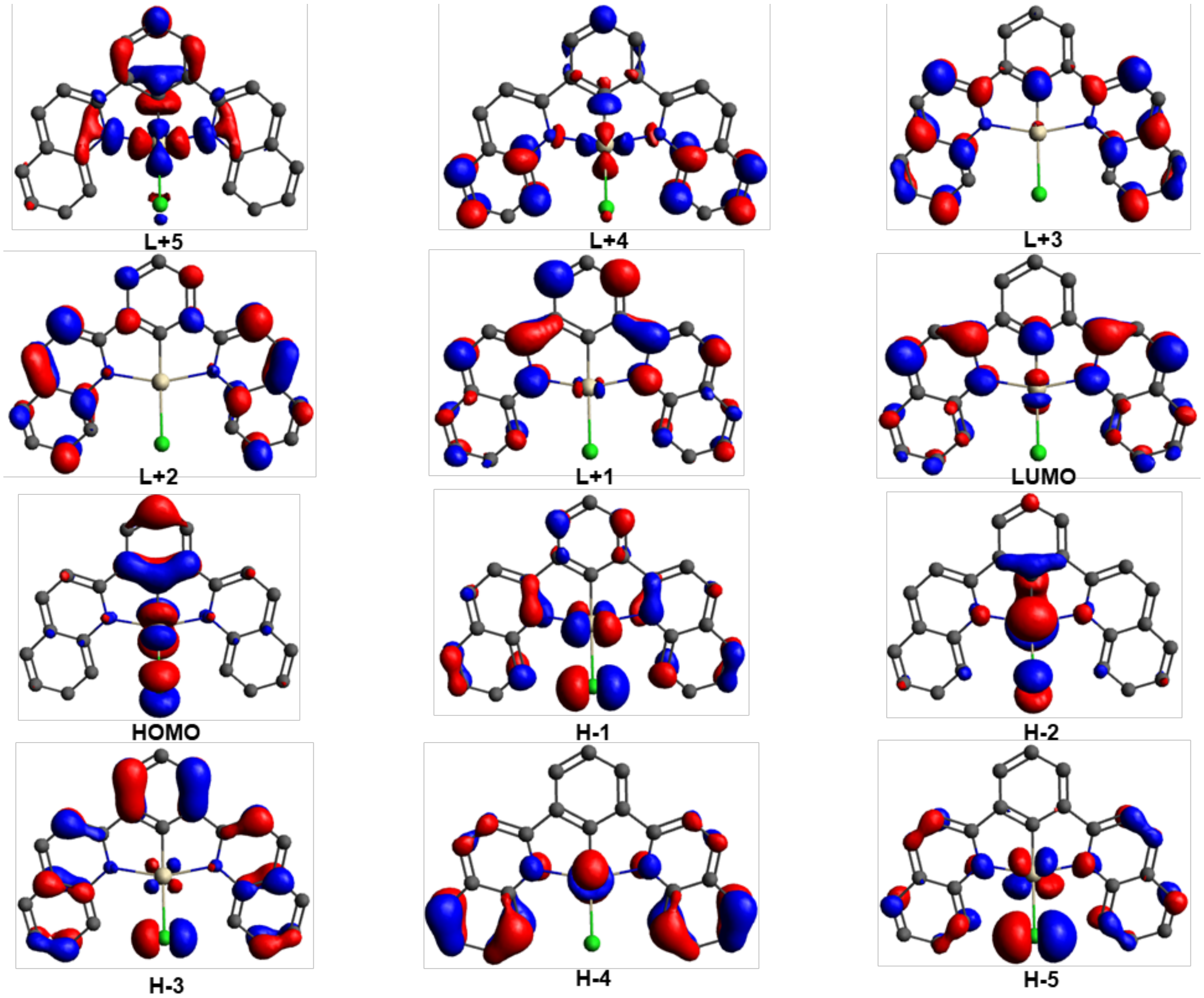

Figure S7. Expanded selection of molecular orbitals of $^{2\text{-Q}}$**L**PtCl (isosurface = 0.05).

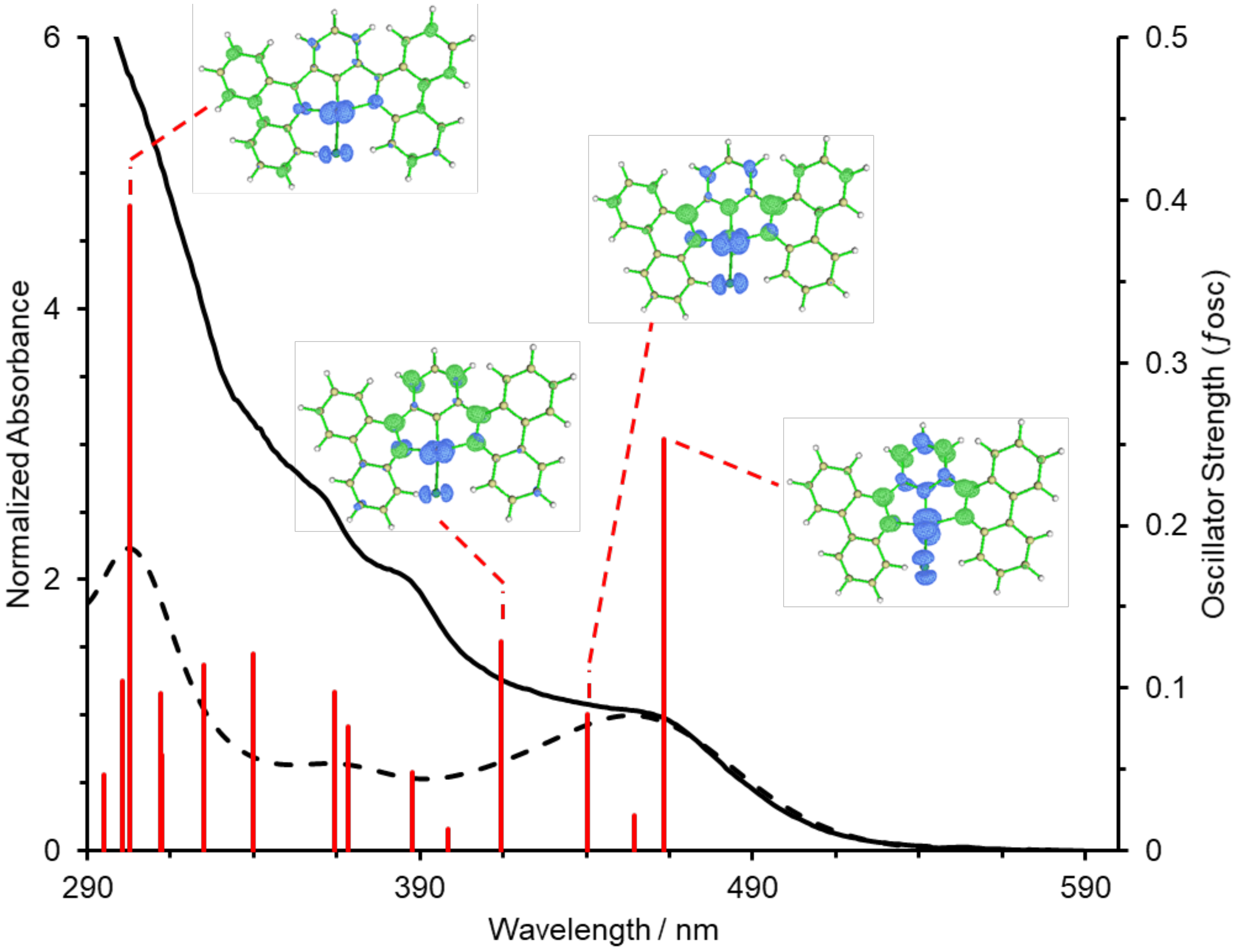

Figure S8. Comparison of the experimental (-) with the computed TD-DFT spectra (---), and vertical energy transitions (red solid line) for **$^{6\text{-}P}$L**PtCl in dichloromethane with generated electron-hole density maps showing the character of selected major transitions (RIJCOSX-ZORA-SMD-M06/def2-TZVP+SARC/J-ZORA-TZVP//SMD-M06L/def2-SVP; isosurface = 0.002). Only transitions with oscillator strength > 0.013 are shown.

Table S4. TD-DFT calculated electronic transitions along with their corresponding excitation energies and oscillator strengths (f_{osc} > 0.013) for **$^{6\text{-}P}$L**PtCl (contributions > 10%).

**No.**	**E / nm**	f_{osc}	**Composition**
1	463.1	0.2530	H-1→L (54%), H→L+1 (24%)
2	454.3	0.0215	H-1→L+1 (50%), H→L (28%)
3	440.2	0.0837	H→L+1 (66%), H-1→L (17%)
4	414.5	0.1290	H→L (66%), H-1→L+1 (22%)
5	398.4	0.0137	H-2→L+1 (62%), H-5→L+1 (14%)
6	387.7	0.0486	H-3→L+1 (41%), H-2→L (25%), H-4→L (18%)
7	368.4	0.0763	H-3→L (45%), H-4→L+1 (30%)
11	340.0	0.1208	H-3→L+2 (16%), H-3→L (12%)
13	325.0	0.1143	H-6→L (15%), H-7→L (13%), H-5→L+1 (12%), H-4→L+1 (11%)
15	312.3	0.0593	H-5→L (26%), H-6→L+1 (16%), H-3→L+1 (19%)
19	302.7	0.3968	H-1→L+2 (25%)
20	300.7	0.1044	H-1→L+7 (23%), H-1→L+3 (10%), H-4→L (10%)
22	295.2	0.0468	H-4→L (24%), H-6→L+1 (12%), H-3→L+1 (10%), H-1→L+7 (10%)

Table S5. Fragment contributions (%) to the frontier MOs of **$^{6\text{-}P}$L**PtCl using Hirshfeld[15] atomic population method.[a]

**MO**	**E (eV)**	**Pt**	**Cl**	**C=N$_1$**	**C=N$_2$**	**Ar(N1)**	**Ar(N2)**	**Ar$_{phenyl}$**
L+2	-1.35	0	0	1	2	34	60	2
L+1	-2.38	9	2	16	15	22	21	14
L	-2.45	2	0	14	15	18	19	31
H	-6.11	29	13	3	3	10	10	33
H-1	6.22	21	14	8	8	17	17	15
H-2	-6.67	34	16	5	4	19	14	9

[a]RIJCOSX-ZORA-SMD-M06/def2-TZVP+SARC/J-ZORA-TZVP//SMD-M06L/def2-SVP

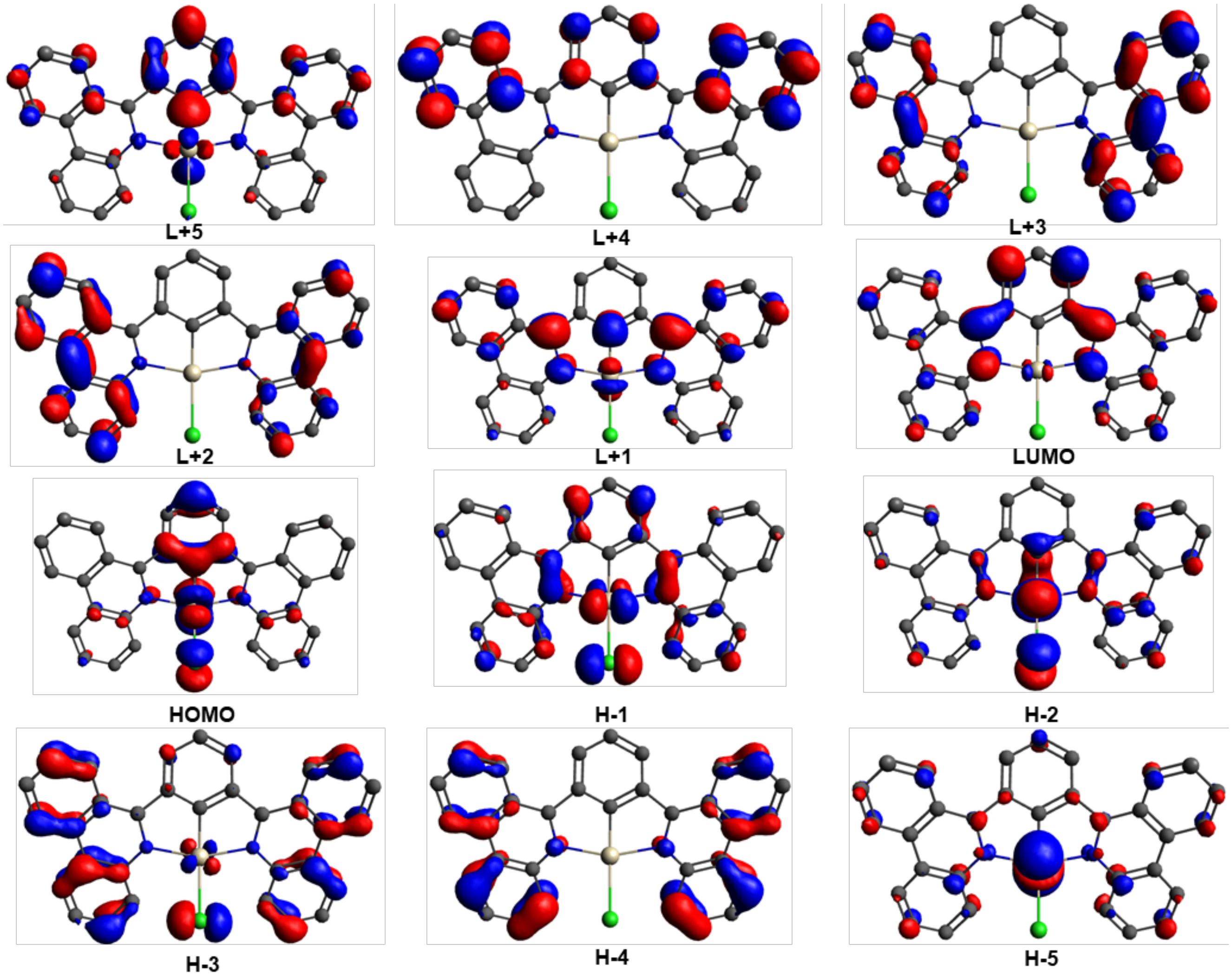

Figure S9. Expanded selection of molecular orbitals of $^{\mathbf{6\text{-}P}}$**L**PtCl (isosurface = 0.05).

Table S6. DFT (SMD-M06L/def2-SVP) optimized ground state and lowest excited triplet state bond lengths (Å) and angles (°).

Complex	State	Pt-N(1)	Pt-N(2)	Pt-C	Pt-Cl	C-Pt-Cl	N(1)-Pt-N(2)	C=N(1)	C=N(2)
$^{2\text{-}Q}$**L**PtCl	S_0	2.123	2.122	1.917	2.519	159.0	158.7	1.359	1.359
	T_1	2.098	2.096	1.926	2.450	136.6	161.1	1.377	1.377
$^{6\text{-}P}$**L**PtCl	S_0	2.103	2.103	1.907	2.518	158.8	158.5	1.349	1.349
	T_1	2.027	2.121	1.924	2.497	162.8	157.9	1.434	1.349

Table S7. Calculated photophysical parameters from single point calculations.[a]

Compound	$E_{adiabatic}$ (T_1-S_0)	$E_{vert,phos}$ (T_1-T_1@S_0)[b]	λ_T (T_1@S_0-S_0)[c]
$^{2\text{-}Q}$**L**PtCl	2.28	1.84	0.44
$^{6\text{-}P}$**L**PtCl	1.94	1.61	0.34

[a]RIJCOSX-ZORA-SMD-M06/def2-TZVP+SARC/J-ZORA-TZVP//SMD-M06L/def2-SVP

[b] T_1@S_0 = Energy from S_0 single point calculation at the T_1 optimized geometry

Supporting Spectroscopic Figures

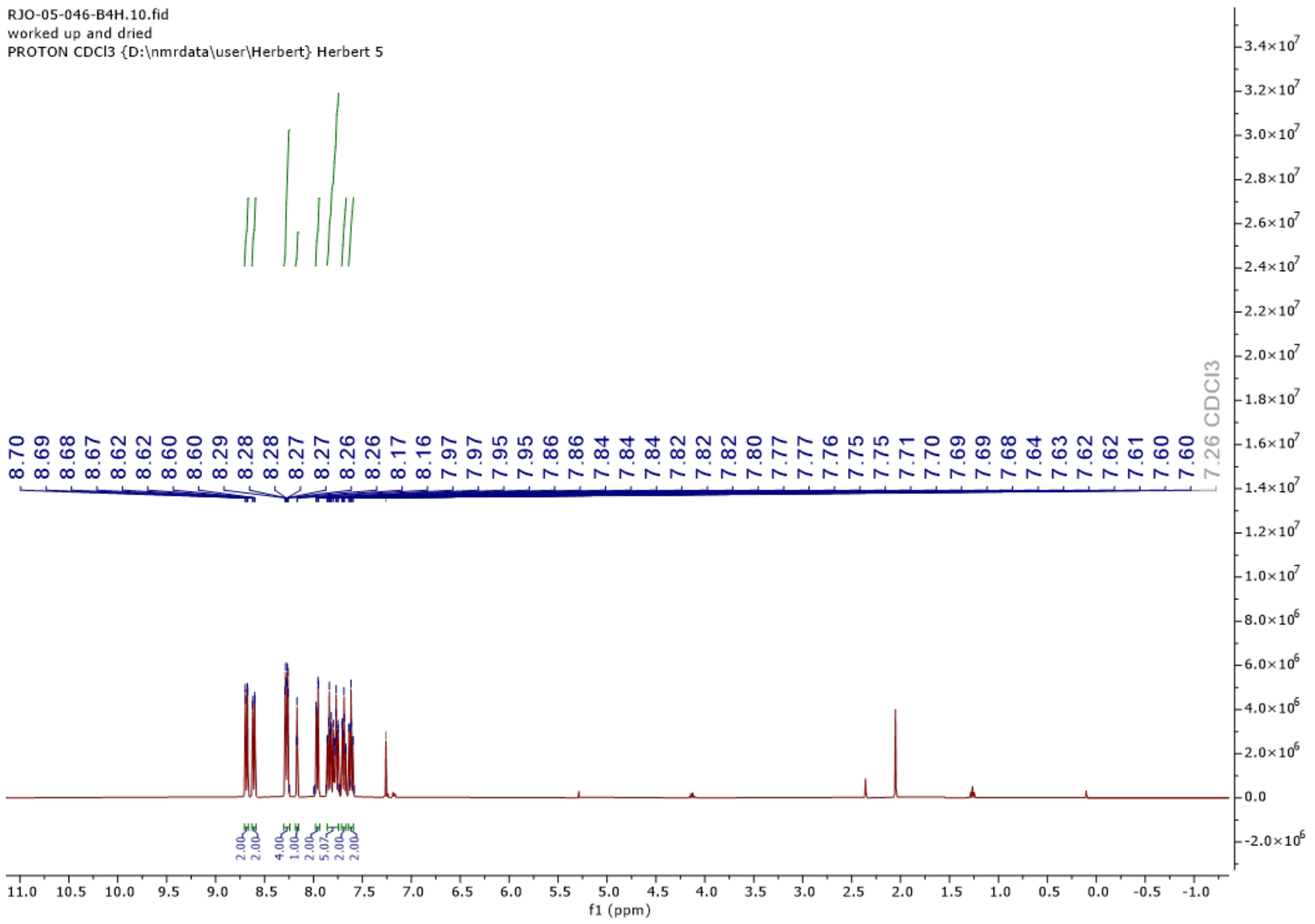

Figure S10. ^{1}H NMR ($CDCl_3$, 400 MHz, 22°C) of **$^{6\text{-}P}$LH.**

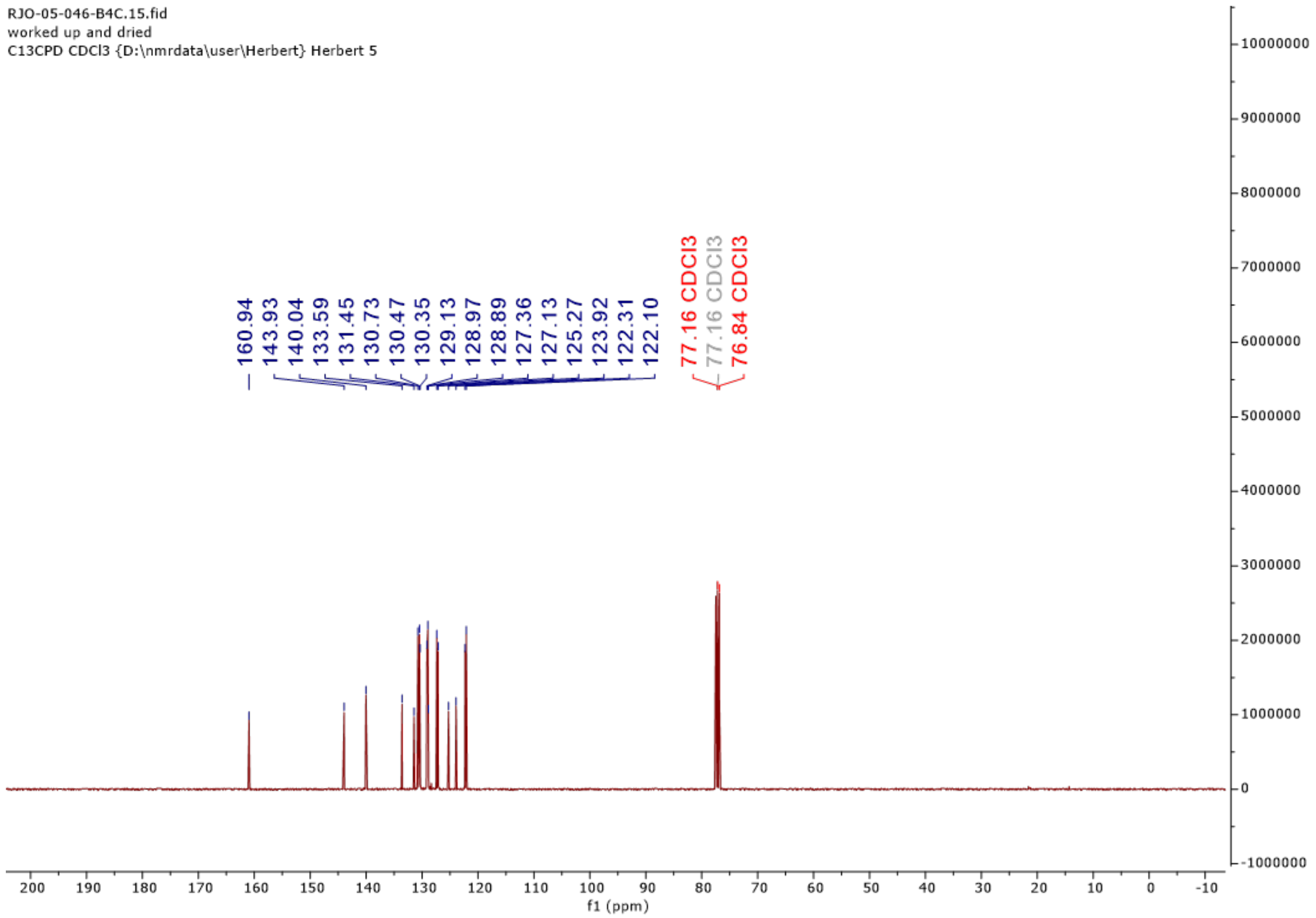

Figure S11. $^{13}C\{H\}$ NMR ($CDCl_3$, 101 MHz, 22 °C) of **$^{6\text{-}P}$L**H.

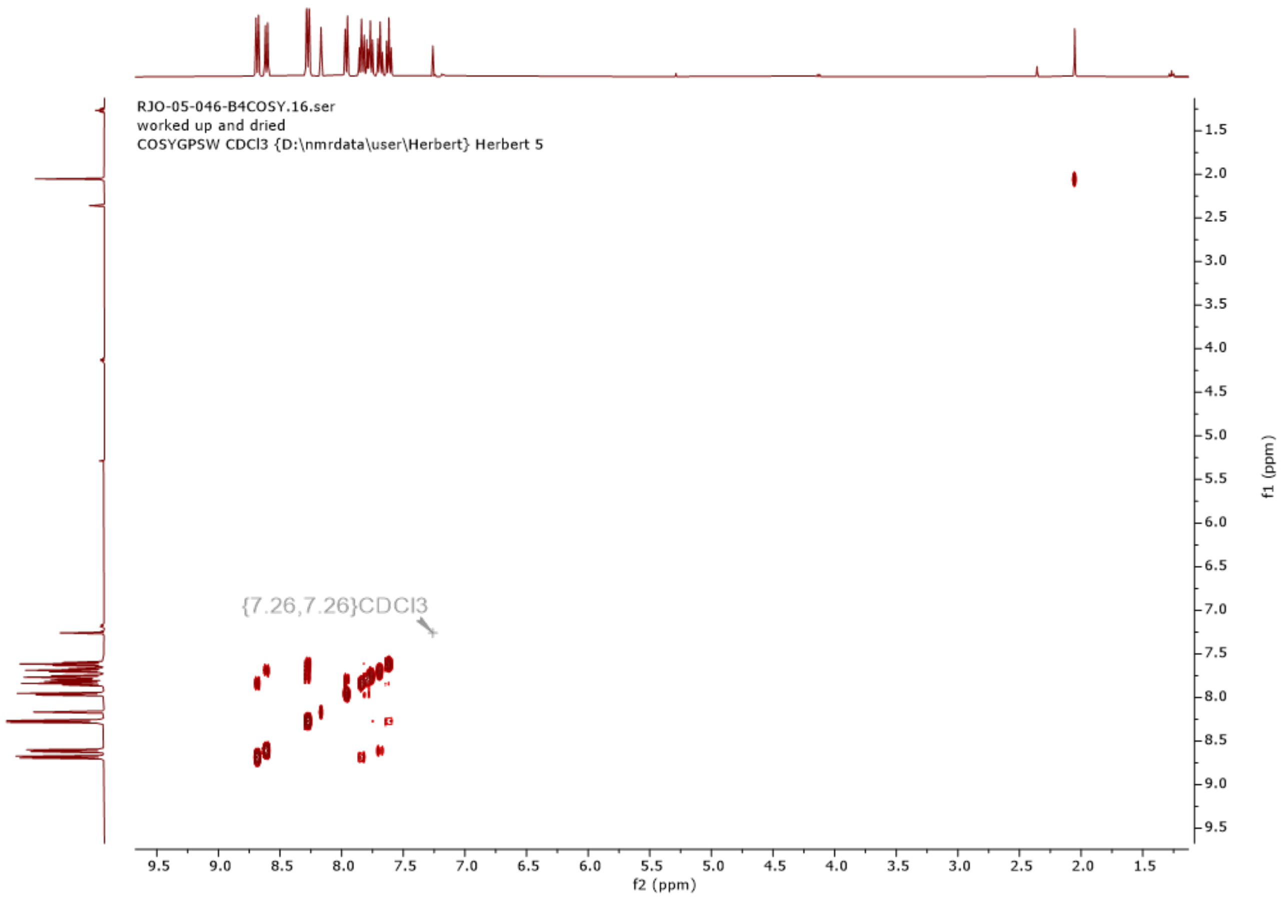

Figure S12. ^{1}H-^{1}H COSY NMR of $^{6\text{-}P}$**L**H.

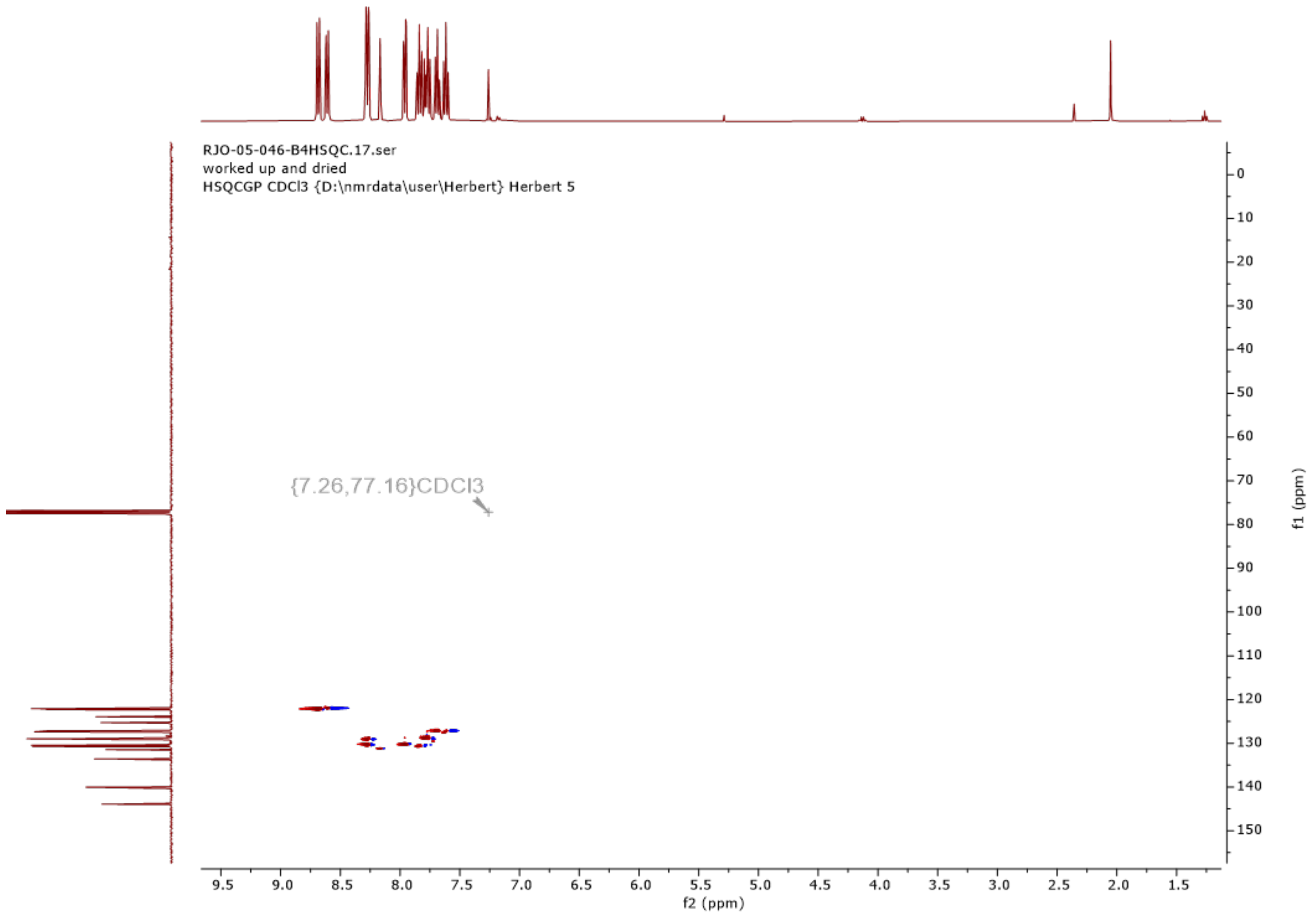

Figure S13. $^{13}C\{H\}$-^{1}H HSQC NMR of $^{\mathbf{6\text{-}P}}$**L**H.

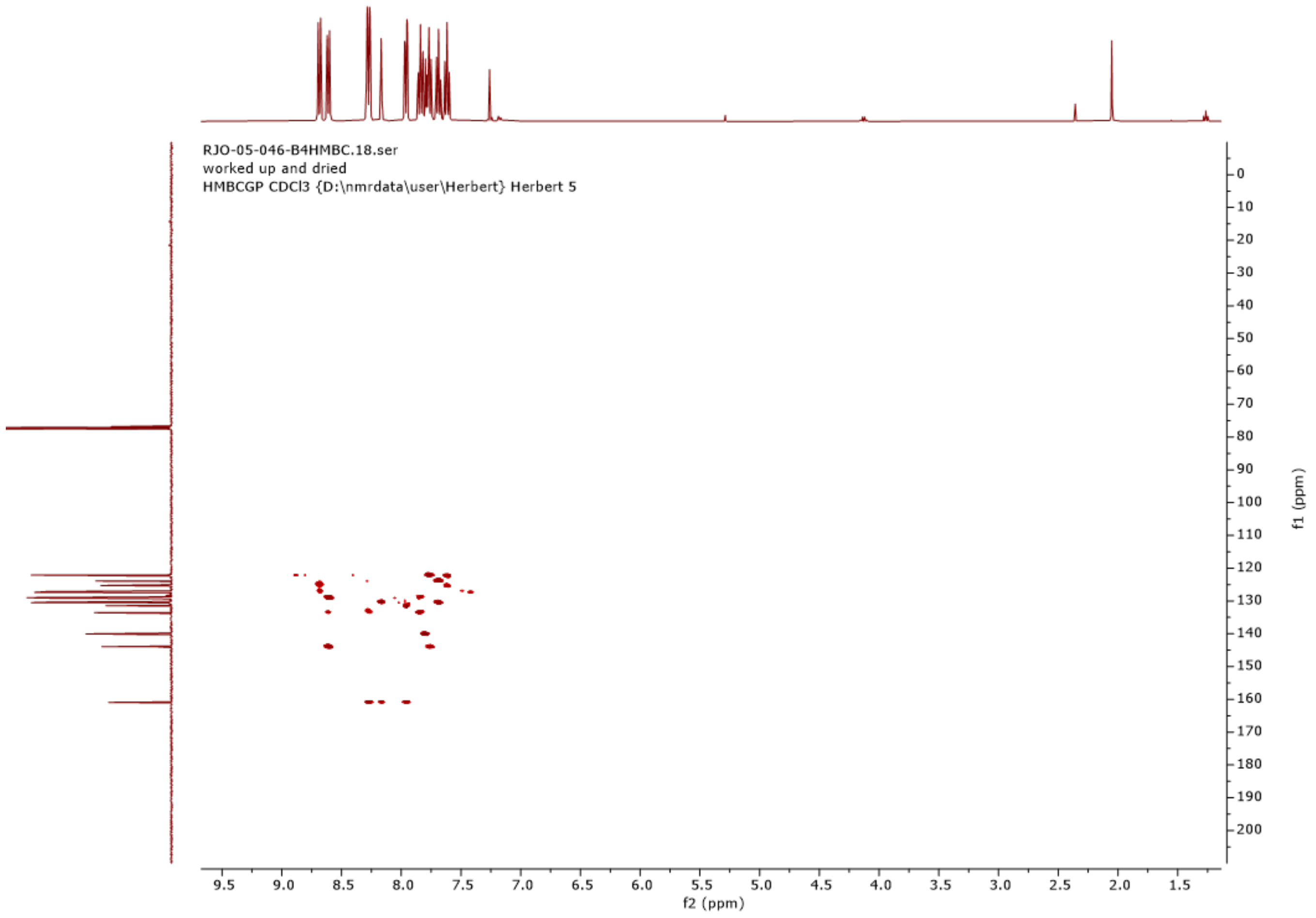

Figure S14. $^{13}C\{H\}$-^{1}H HMBC NMR of **$^{6\text{-}P}$L**H.

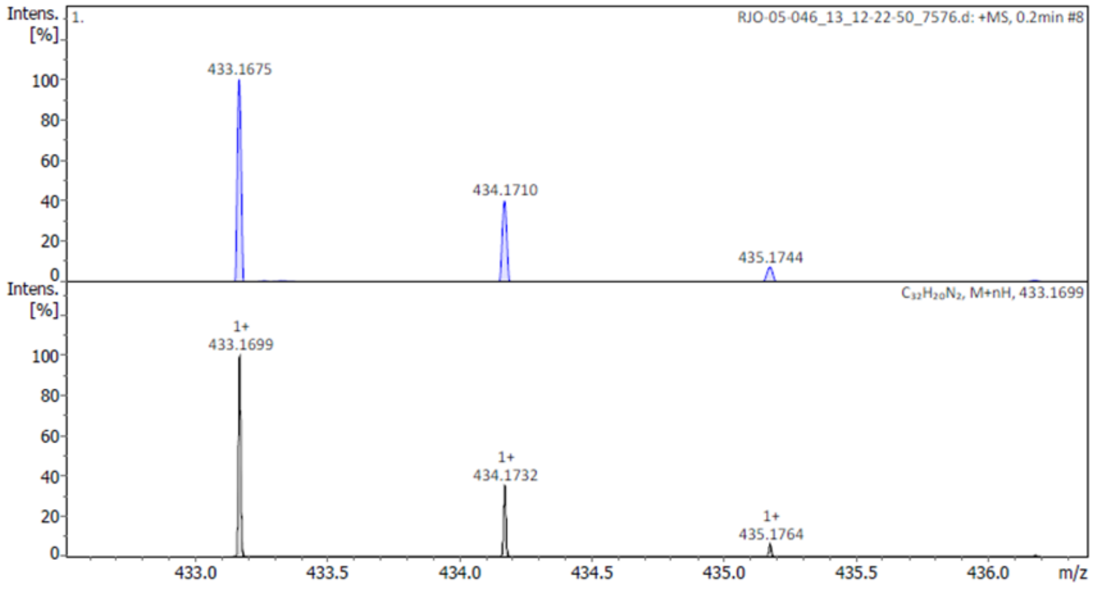

Figure S15. HRMS (APCI-TOF) of **$^{6\text{-}P}$L**H.

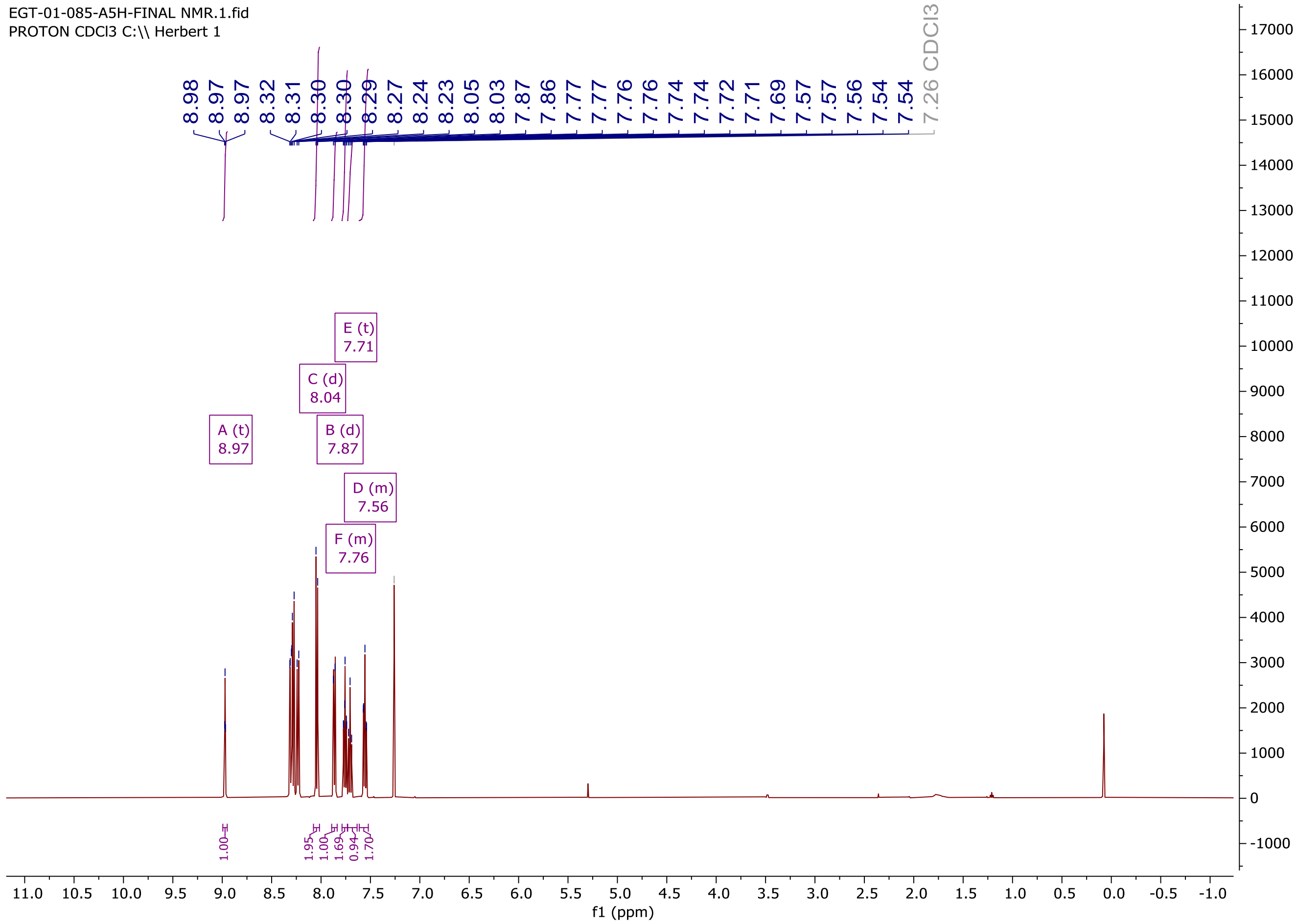

Figure S16. ^{1}H NMR ($CDCl_3$, 500 MHz, 22°C) of **$^{2\text{-}Q}$LH.**

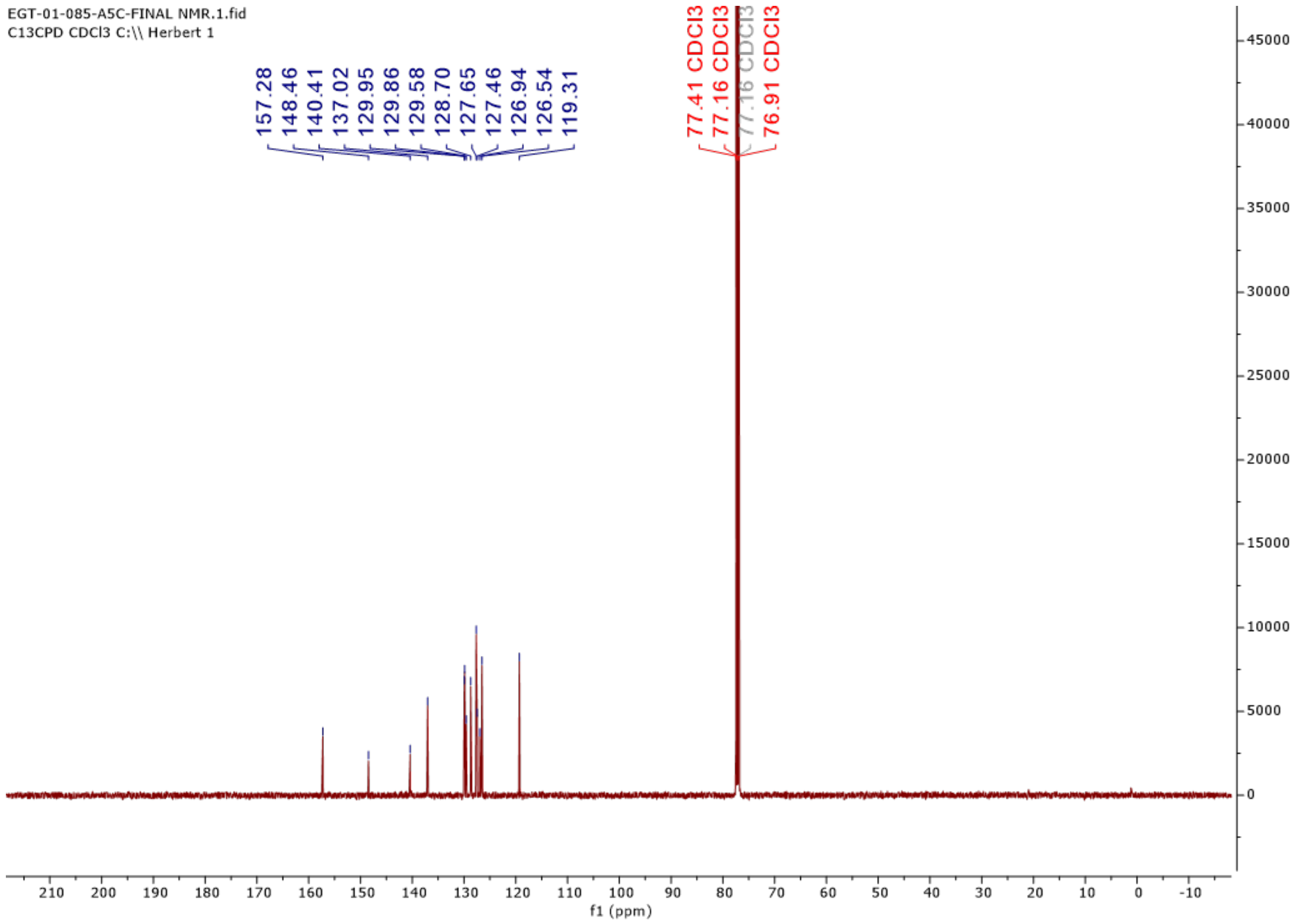

Figure S17. $^{13}C\{H\}$ NMR ($CDCl_3$, 126 MHz, 22 °C) of **$^{2\text{-}Q}$LH.**

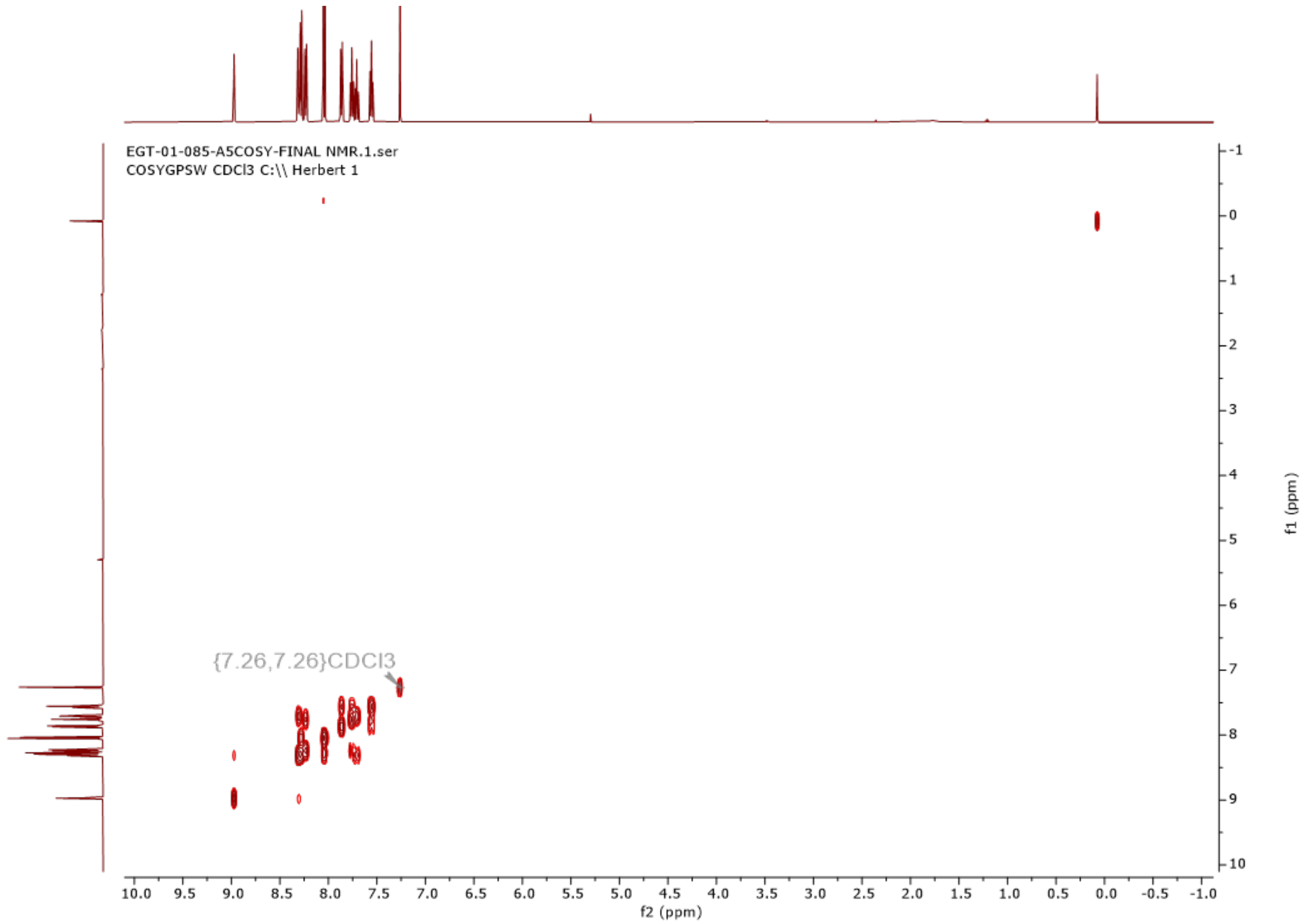

Figure S18. ^{1}H-^{1}H COSY NMR of **$^{2\text{-}Q}$L**H.

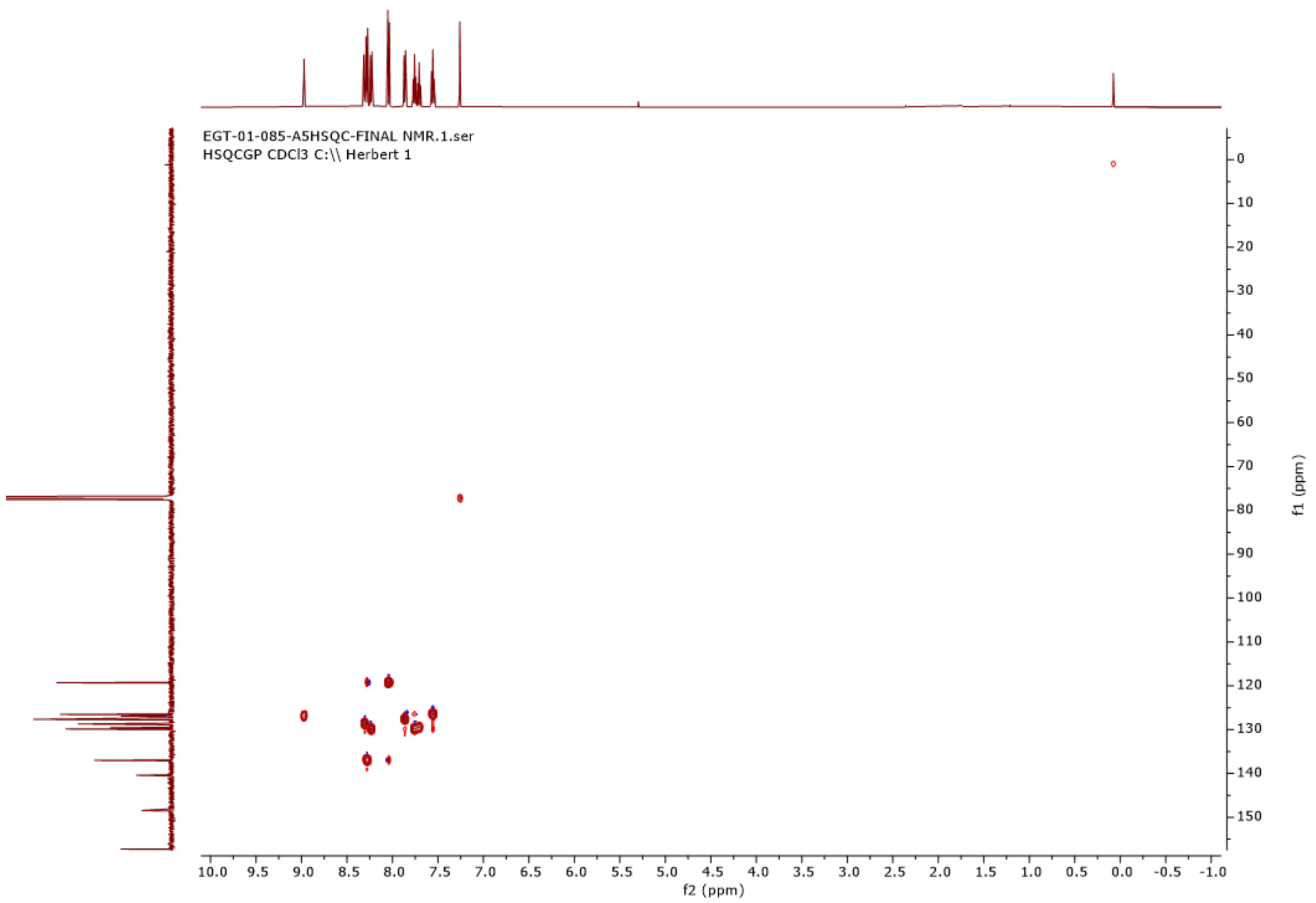

Figure S19. $^{13}C\{H\}$-^{1}H HSQC NMR of **$^{2\text{-}Q}$L**H.

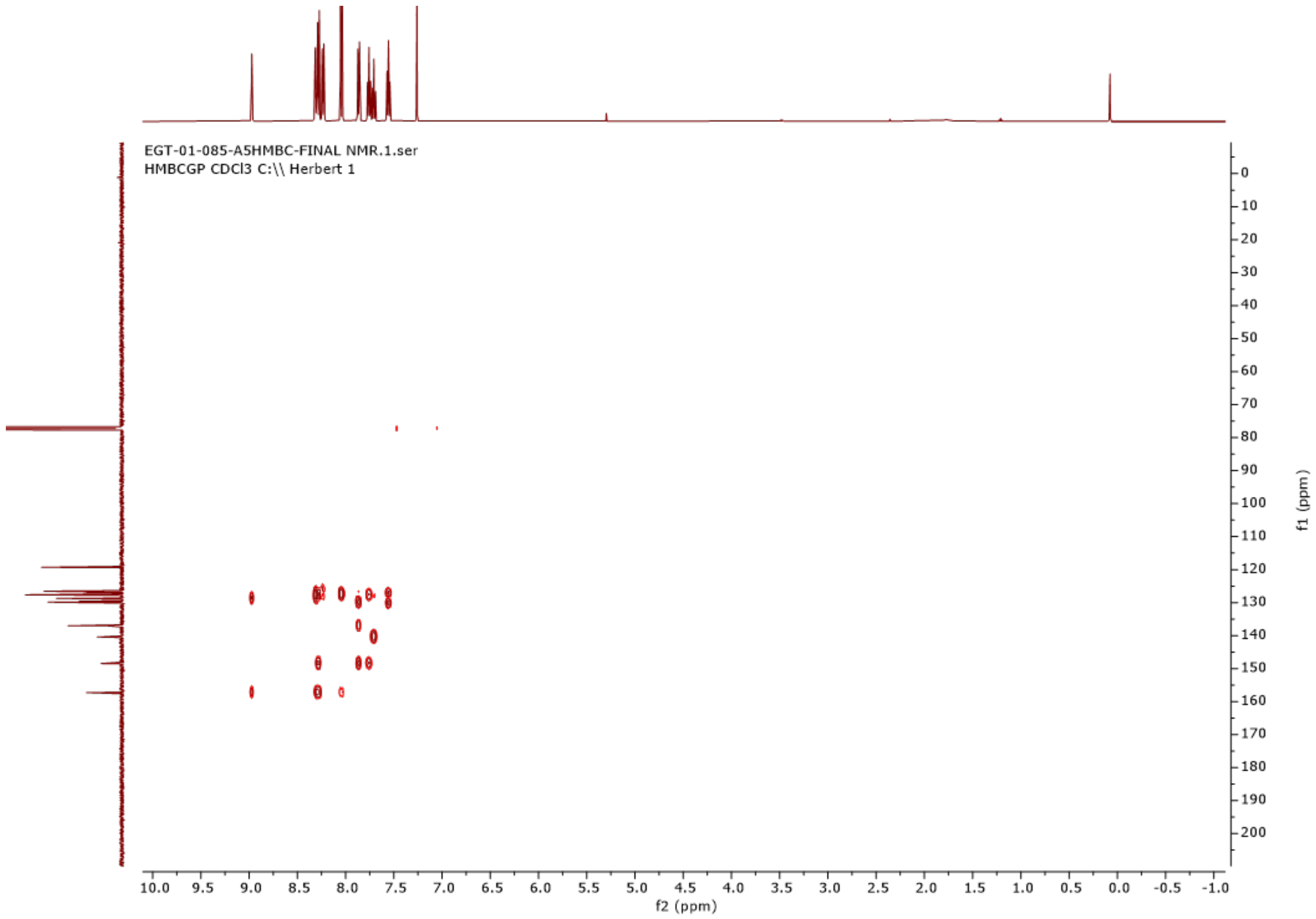

Figure S20. $^{13}C\{H\}$-^{1}H HMBC NMR of **$^{2\text{-}Q}$L**H.

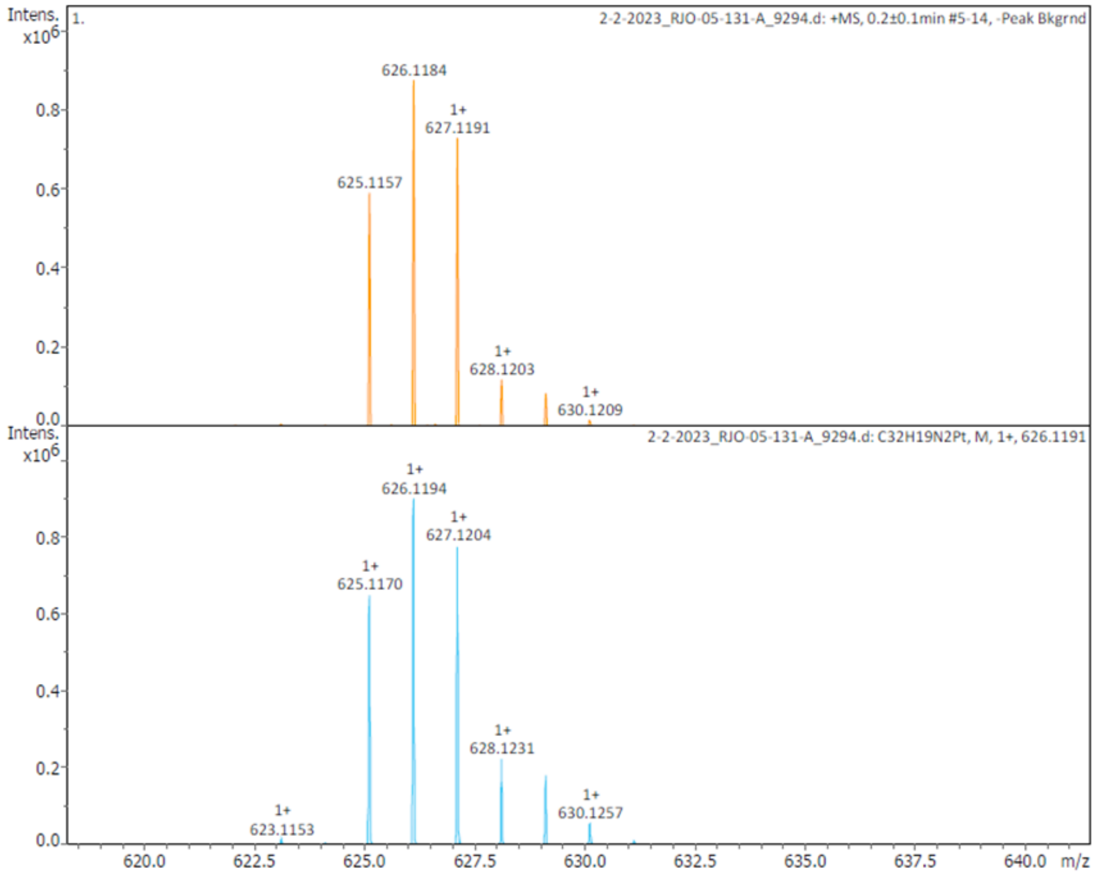

Figure S21. HRMS (APCI-TOF) of **$^{6\text{-}P}$L**PtCl.

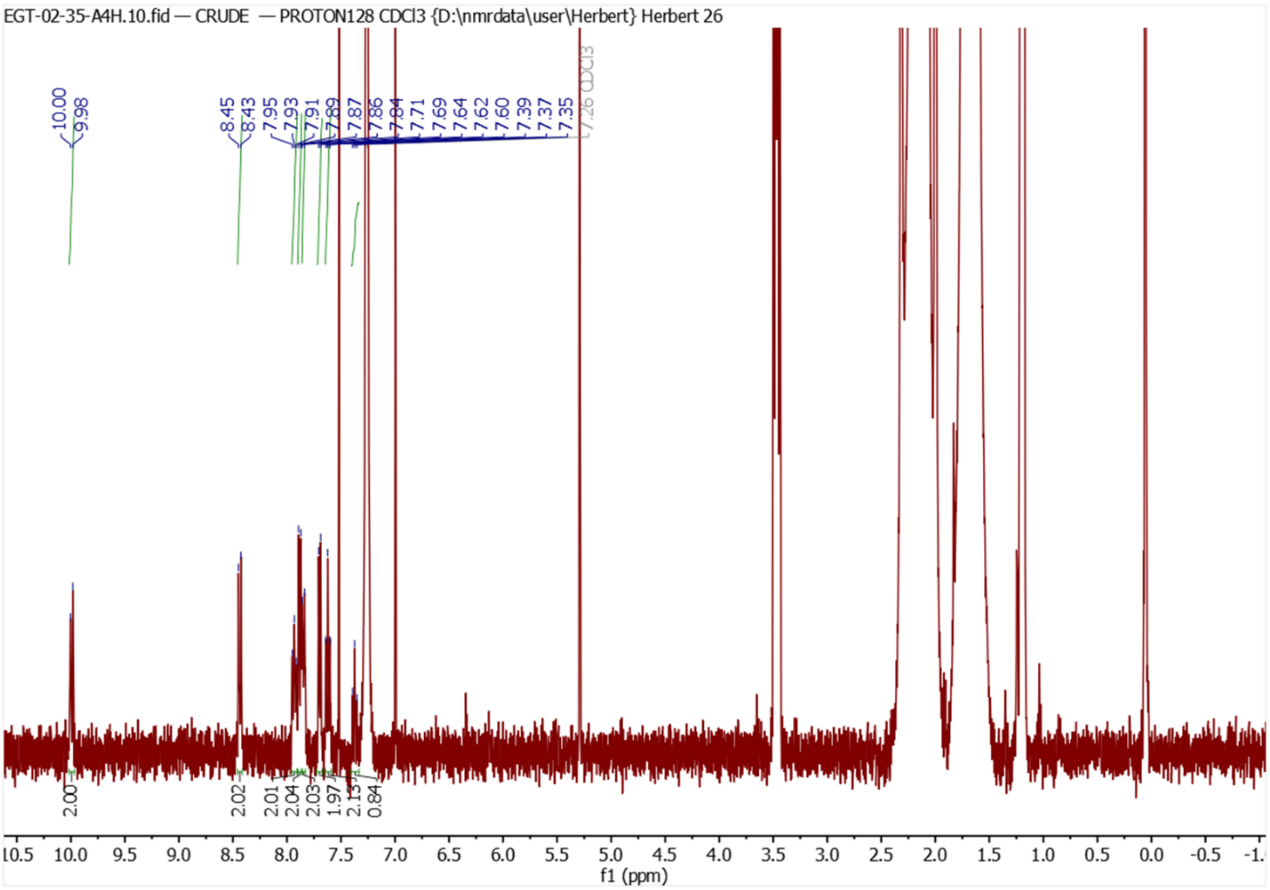

Figure S22. ^{1}H NMR ($CDCl_3$, 400 MHz, 22°C) of **2-QLPtCl.**

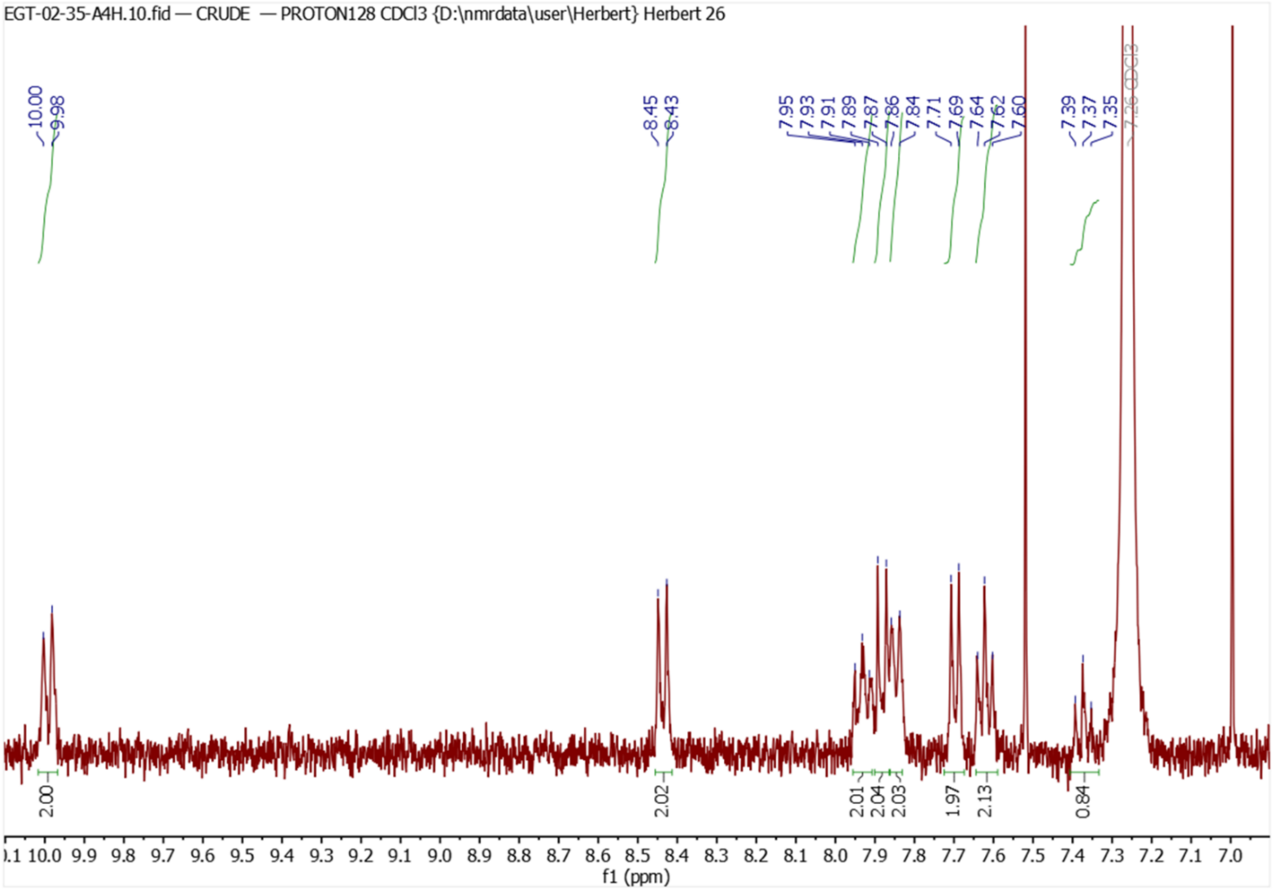

Figure S23. ^{1}H NMR ($CDCl_3$, 400 MHz, 22°C) zoom into aromatic region of $^{2\text{-}Q}$**L**PtCl**.**

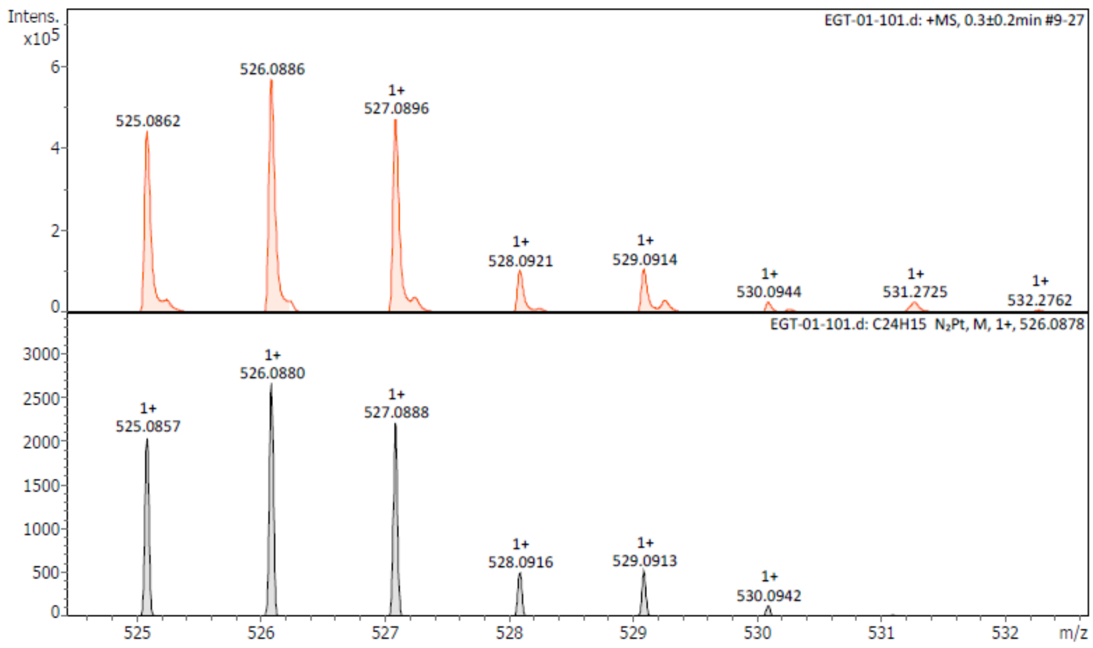

Figure S24. HRMS (APCI-TOF) of $^{2\text{-}Q}$**L**PtCl.

ENERGIES AND REACTION COORDINATES

[2-Q]L PtCl, $S = 0$

```
--------------------------------
INTERNAL COORDINATES (ANGSTROEM)
--------------------------------
```

Pt	0	0	0	0.000000000000	0.00000000	0.00000000
Cl	1	0	0	2.519389797333	0.00000000	0.00000000
N	1	2	0	2.123076777313	100.25310165	0.00000000
N	1	2	3	2.122155399176	100.87795317	182.50825135
C	4	1	2	1.359436555539	112.30520970	150.34579451
C	3	1	2	1.374922579595	128.36376642	31.00829986
C	3	1	2	2.376185367371	78.81368531	206.46117474
C	6	3	1	1.434152160825	120.59448116	186.55650825
C	7	3	1	1.404332598360	82.28907599	353.11042808
C	8	6	3	2.407191581529	88.76776995	356.13478265
C	8	6	3	1.415223096808	119.45979374	179.12651433
H	11	8	6	1.092533790990	118.24835498	180.92971251
C	5	4	1	1.411947031089	121.49082388	185.00488580
C	10	8	6	1.369542833038	30.71819951	177.67708785
C	4	1	2	1.375281714626	128.28165876	328.63185072
C	9	7	3	2.413660634173	91.33146321	176.50619809
H	16	9	7	1.092855477161	151.55910236	182.76381313
C	11	8	6	1.377146328658	120.66978471	1.83258628
H	18	11	8	1.091797505636	120.33474417	179.57309538
C	6	3	1	1.413156712516	120.80989739	8.68418792
H	20	6	3	1.089004987248	118.83794743	359.57508775

C	16	9	7	1.402965806998	30.72932769	183.71260708
C	16	9	7	1.397708712012	88.96777386	2.67292905
H	23	16	9	1.091659693097	119.34679466	179.01581715
C	3	1	2	1.359471245978	112.21514562	210.25819479
C	15	4	1	2.423402037924	150.25359075	352.42116083
H	26	15	4	1.092206384832	149.39306764	359.72529723
C	13	5	4	1.369411087315	120.06727917	358.21343699
C	28	13	5	1.414168695006	119.63157873	357.20741949
C	20	6	3	1.379455569558	120.36529517	179.76914004
H	30	20	6	1.092200329337	119.25034771	180.48305916
C	23	16	9	1.397413211492	121.35629285	357.64265529
H	32	23	16	1.092973112478	119.47918033	182.07736646
C	29	28	13	1.415331528490	122.05050039	184.28444653
H	34	29	28	1.092420188016	118.23192710	357.66782884
C	26	15	4	1.379634980962	30.20776699	0.77739032
H	36	26	15	1.088868906725	120.80025235	179.87506585
C	34	29	28	1.377035263911	120.67794665	176.63494546
H	38	34	29	1.091928406544	120.35828342	180.50944586
H	13	5	4	1.090045521300	118.88183182	178.55511195
H	28	13	5	1.092069709748	120.91754826	179.22208060
H	10	8	6	1.090254469382	151.71082091	182.75277744
H	14	10	8	1.092202739503	120.92399616	178.25491086

[2-Q]**L**PtCl, $S = 1$

INTERNAL COORDINATES (ANGSTROEM)

Pt	0	0	0	0.000000000000	0.00000000	0.00000000
Cl	1	0	0	2.449719186016	0.00000000	0.00000000
N	1	2	0	2.097794933211	97.46169676	0.00000000
N	1	2	3	2.096426231601	98.36514673	190.28610267
C	4	1	2	1.376880789071	112.82646973	132.49962974
C	3	1	2	1.374663773550	127.28718067	46.60652884
C	3	1	2	2.376401949685	79.09526525	224.38639211
C	6	3	1	1.435471301569	120.34393796	187.11028243
C	7	3	1	1.405826176862	82.95869446	359.22979709
C	3	1	2	2.418143504660	142.33214591	232.22412679
C	8	6	3	1.413700132259	118.79141276	178.24078808
H	11	8	6	1.092734883205	118.17534664	180.73247908
C	5	4	1	1.401211544784	121.05189406	184.93998582
C	10	3	1	1.379449051433	91.15766288	171.70779638
C	4	1	2	1.374627157970	127.22683894	313.38991656
C	9	7	3	2.384350131409	93.83435540	183.39837760
H	16	9	7	1.092705730774	152.73668080	179.48579257
C	11	8	6	1.382593501349	121.06977482	1.41878170
H	18	11	8	1.091923089746	120.18608052	179.90119169
C	6	3	1	1.409617805742	120.69386336	8.44032158
H	20	6	3	1.091407152032	118.97531249	1.44569116
C	16	9	7	1.400389340521	31.89889824	181.14383746

C	16	9	7	1.402028053085	87.67568825	0.40160065
H	23	16	9	1.091921868361	118.75279786	180.13314416
C	3	1	2	1.376827280565	112.77646480	227.77244195
C	15	4	1	2.427489570017	150.07821814	353.40358602
H	26	15	4	1.092014762431	149.37652165	358.28669233
C	13	5	4	1.379264992012	120.34256213	359.96587528
C	28	13	5	1.419986817591	119.62230082	357.29990707
C	20	6	3	1.384292606605	120.62678665	181.27411201
H	30	20	6	1.092002152142	119.42167447	180.00709942
C	7	3	1	1.400659622299	160.32448494	189.90211974
H	32	7	3	1.092938690733	120.75468707	348.88107665
C	29	28	13	1.413404595653	122.69355031	182.70314715
H	34	29	28	1.092737859679	118.26464682	358.14496087
C	26	15	4	1.384564817170	29.97749878	358.22805757
H	36	26	15	1.091535379363	120.43285355	180.16304150
C	34	29	28	1.382790490656	121.06487772	177.52995993
H	38	34	29	1.092004419048	120.17248554	180.39122846
H	13	5	4	1.091027055299	118.76079002	179.57476045
H	28	13	5	1.091689482794	120.94023015	178.66879898
H	10	3	1	1.091258777098	147.84229087	352.33527479
H	14	10	3	1.091745123315	120.87473066	181.46984895

$^{6\text{-P}}$**L**PtCl, $S = 0$

INTERNAL COORDINATES (ANGSTROEM)

Pt	0	0	0	0.000000000000	0.00000000	0.00000000
Cl	1	0	0	2.517934902941	0.00000000	0.00000000
N	1	2	0	2.103033049021	100.41350362	0.00000000
N	1	2	3	2.103202916508	100.98937257	181.57792897
C	4	1	2	1.348691463275	112.98495848	146.34657444
C	3	1	2	1.384945461788	126.27746736	36.65054159
C	3	1	2	2.358288039304	79.51211773	205.59878739
C	6	3	1	1.422919947648	121.08161877	185.32654072
C	7	3	1	1.412483800798	81.98148521	354.59230012
C	3	1	2	2.421250180228	142.46078793	226.96891799
C	8	6	3	1.409909687384	118.50693490	183.72040644
H	11	8	6	1.090631074105	119.52691863	179.65054856
C	5	4	1	1.444766152340	120.18528231	195.15069663
C	10	3	1	1.425210039851	90.82582606	168.99027444
C	4	1	2	1.384975179803	126.28024217	324.68152509
C	9	7	3	2.403914086355	92.18179496	184.36081913
H	16	9	7	1.089329798312	152.75463120	175.71075616
C	11	8	6	1.382273863768	121.27322312	0.47016076
H	18	11	8	1.091650613825	119.96641072	179.66481652
C	6	3	1	1.410131403489	119.31143558	9.88083395
H	20	6	3	1.088901892859	118.84589056	356.33398584
C	14	10	3	1.409844085173	118.93596336	186.45184047
H	22	14	10	1.090221386808	119.72642953	180.43534997
C	16	9	7	1.406587866161	31.54086428	190.01852072

C	13	5	4	1.415687763643	122.26184285	162.18426123
H	25	13	5	1.087209729945	120.01652558	357.44495519
C	16	9	7	1.395386356725	88.44157561	6.37582860
H	27	16	9	1.091252696284	118.97226140	174.98531857
C	3	1	2	1.349116504777	112.96357735	214.20922345
C	15	4	1	2.424820622122	148.54016921	346.55636113
H	30	15	4	1.091783165815	149.56373016	4.62022790
C	13	5	4	1.425025224737	118.99323061	346.68182067
C	25	13	5	2.411782760582	90.84915637	180.98442234
H	33	25	13	1.091852359692	149.78502493	180.86337428
C	15	4	1	1.422870431724	121.14271457	173.88249631
C	20	6	3	1.381472596154	120.57351333	175.98348676
H	36	20	6	1.091905193364	119.40840916	180.86402384
C	27	16	9	1.395334756002	121.90800180	354.55284555
H	38	27	16	1.089449546204	118.47827764	180.22505553
C	35	15	4	1.410247249981	118.45604698	176.95578815
H	40	35	15	1.090541019421	119.49946962	179.93276687
C	25	13	5	1.380808152189	121.09462817	181.01199835
H	42	25	13	1.091643703063	119.78217958	178.68789012
C	30	15	4	1.381125505312	30.03259977	5.62759951
H	44	30	15	1.088685048556	120.68080865	180.80580867
C	33	25	13	1.381516604752	90.36738616	2.17365621
H	46	33	25	1.090354288677	119.27464153	178.38290743
C	22	14	10	2.412806778901	90.87298903	2.76074840

H	48	22	14	1.091478605673	149.74484468	174.58513196
C	48	22	14	1.380892734270	90.36249083	359.07068049
H	50	48	22	1.087724239293	118.79579165	175.18936407
C	22	14	10	1.381557517423	121.08711614	2.02282935
H	52	22	14	1.091823806087	119.82818341	179.43727399
C	40	35	15	1.381979003566	121.29037243	359.44648219
H	54	40	35	1.091680875770	119.84436465	180.12377180

$^{6\text{-P}}$**L**PtCl, *S* = 1

```
--------------------------------
INTERNAL COORDINATES (ANGSTROEM)
--------------------------------
```

Pt	0	0	0	0.000000000000	0.00000000	0.00000000
Cl	1	0	0	2.496566487148	0.00000000	0.00000000
N	1	2	0	2.027065829591	97.43250465	0.00000000
N	1	2	3	2.120609937862	104.45260383	177.19461374
C	4	1	2	1.349404718505	112.76018963	148.61196978
C	3	1	2	1.363516818928	127.84726086	42.34197283
C	3	1	2	2.382000156311	80.59708304	201.80346508
C	6	3	1	1.434388945953	120.47986281	187.18876136
C	7	3	1	1.422473764839	80.67571484	353.90322854
C	3	1	2	2.465880343540	140.49718104	228.20303719
C	8	6	3	1.396210068418	118.15655773	179.56575072
H	11	8	6	1.091156762792	119.30809494	180.25220978

C	5	4	1	1.443537956120	119.96985715	196.08649629
C	10	3	1	1.426431490256	89.48561744	167.34007278
C	4	1	2	1.382892549118	126.19100459	328.17920613
C	9	7	3	2.406056663496	92.52119765	183.10263786
H	16	9	7	1.089091365364	152.32126092	172.46125735
C	11	8	6	1.395751516252	121.88308911	0.42634063
H	18	11	8	1.091590655717	119.78912385	179.29204455
C	6	3	1	1.418277673618	120.26780232	7.24198774
H	20	6	3	1.089697406719	118.43484325	1.88131476
C	14	10	3	1.398479590977	119.02752418	189.35078297
H	22	14	10	1.091341049927	119.66389404	179.97687396
C	9	7	3	1.400170620834	123.70679641	177.98650577
C	13	5	4	1.415473331380	122.19142730	161.87089996
H	25	13	5	1.087541207279	119.91756296	356.76505991
C	16	9	7	1.400507979344	88.45552869	4.64455624
H	27	16	9	1.091832499575	118.61528262	175.45553859
C	7	3	1	1.423707026413	33.68414561	162.04657047
C	15	4	1	2.424438417256	148.66270459	347.11491035
H	30	15	4	1.091854465170	149.51754573	4.57088847
C	13	5	4	1.425631749387	118.94132437	345.89832384
C	25	13	5	2.411143001590	90.74454014	180.32613498
H	33	25	13	1.091900552649	149.76674693	180.88506626
C	15	4	1	1.423296617075	121.02434265	173.52682069
C	20	6	3	1.384088448300	120.79699741	180.27320349

H	36	20	6	1.091855984872	119.52214439	180.94246154
C	27	16	9	1.390612010202	122.38609965	356.34839771
H	38	27	16	1.091421136222	119.28835777	178.28428050
C	35	15	4	1.409601878962	118.41950085	177.01968961
H	40	35	15	1.090595472201	119.49013311	179.87990630
C	25	13	5	1.380484653912	121.02328883	180.36570276
H	42	25	13	1.091576962534	119.77435202	178.56448635
C	30	15	4	1.381301400333	30.04877219	5.20992861
H	44	30	15	1.089301833143	120.71034918	180.99972702
C	33	25	13	1.381610874413	90.45270751	2.22000142
H	46	33	25	1.090384263991	119.29706746	178.34436213
C	22	14	10	2.413263997275	91.26408397	0.68486374
H	48	22	14	1.092076057204	150.37120217	176.76339794
C	48	22	14	1.388945307073	89.93752279	0.11629359
H	50	48	22	1.090316759940	119.25946341	176.03970705
C	22	14	10	1.394802740799	121.42625541	0.29629375
H	52	22	14	1.091815332479	119.80103767	179.48996801
C	40	35	15	1.382863400807	121.28958895	359.80705167
H	54	40	35	1.091659976672	119.80790603	179.95579791

References

(1) Suzuki, K.; Kobayashi, A.; Kaneko, S.; Takehira, K.; Yoshihara, T.; Ishida, H.; Shiina, Y.; Oishi, S.; Tobita, S. Reevaluation of Absolute Luminescence Quantum Yields of Standard Solutions Using a Spectrometer with an Integrating Sphere and a Back-Thinned CCD Detector. *Phys. Chem. Chem. Phys.* **2009**, *11*, 9850–9860.

(2) Murov, S. L.; Carmichael, I.; Hug, G. L. *Handbook of Photochemistry, 2nd Ed.*; Marcel Dekker: New York, 1993.

(3) Neese, F. The ORCA Program System. *WIREs Comput. Mol. Sci.* **2012**, *2*, 73–78.

(4) Neese, F. Software Update: The ORCA Program System—Version 5.0. *WIREs Comput. Mol. Sci.* **2022**, *12*, e1606.

(5) Zhao, Y.; Truhlar, D. G. A New Local Density Functional for Main-Group Thermochemistry, Transition Metal Bonding, Thermochemical Kinetics, and Noncovalent Interactions. *J. Chem. Phys.* **2006**, *125*, 194101.

(6) Andrae, D.; Häußermann, U.; Dolg, M.; Stoll, H.; Preuß, H. Energy-Adjustedab Initio Pseudopotentials for the Second and Third Row Transition Elements. *Theoret. Chim. Acta* **1990**, *77*, 123–141.

(7) Weigend, F.; Ahlrichs, R. Balanced Basis Sets of Split Valence, Triple Zeta Valence and Quadruple Zeta Valence Quality for H to Rn: Design and Assessment of Accuracy. *Phys. Chem. Chem. Phys.* **2005**, *7*, 3297–3305.

(8) Marenich, A. V.; Cramer, C. J.; Truhlar, D. G. Universal Solvation Model Based on Solute Electron Density and on a Continuum Model of the Solvent Defined by the Bulk Dielectric Constant and Atomic Surface Tensions. *J. Phys. Chem. B* **2009**, *113*, 6378–6396.

(9) Neese, F.; Wennmohs, F.; Hansen, A.; Becker, U. Efficient, Approximate and Parallel Hartree–Fock and Hybrid DFT Calculations. A 'Chain-of-Spheres' Algorithm for the Hartree–Fock Exchange. *Chem. Phys.* **2009**, *356*, 98–109.

(10) Weigend, F. Accurate Coulomb-Fitting Basis Sets for H to Rn. *Phys. Chem. Chem. Phys.* **2006**, *8*, 1057–1065.

(11) Pantazis, D. A.; Neese, F. All-Electron Scalar Relativistic Basis Sets for the Lanthanides. *J. Chem. Theory Comput.* **2009**, *5*, 2229–2238.

(12) Pantazis, D. A.; Chen, X.-Y.; Landis, C. R.; Neese, F. All-Electron Scalar Relativistic Basis Sets for Third-Row Transition Metal Atoms. *J. Chem. Theory Comput.* **2008**, *4*, 908–919.

(13) Pantazis, D. A.; Neese, F. All-Electron Scalar Relativistic Basis Sets for the 6p Elements. *Theor. Chem. Acc.* **2012**, *131*, 1292–1298.

(14) Pantazis, D. A.; Neese, F. All-Electron Scalar Relativistic Basis Sets for the Actinides. *J. Chem. Theory Comput.* **2011**, *7*, 677–684.

(15) Hirshfeld, F. L. Bonded-Atom Fragments for Describing Molecular Charge Densities. *Theoret. Chim. Acta* **1977**, *44*, 129–138.

(16) Lu, T.; Chen, F. Multiwfn: A Multifunctional Wavefunction Analyzer. *J. Comput. Chem.* **2012**, *33*, 580–592.

(17) Hanwell, M. D.; Curtis, D. E.; Lonie, D. C.; Vandermeersch, T.; Zurek, E.; Hutchison, G. R. Avogadro: An Advanced Semantic Chemical Editor, Visualization, and Analysis Platform. *J. Cheminform.* **2012**, *4*, 17.

(18) Allouche, A.-R. Gabedit—A Graphical User Interface for Computational Chemistry Softwares. *J. Comput. Chem.* **2011**, *32*, 174–182.

(19) Mandapati, P.; Braun, J. D.; Lozada, I. B.; Williams, J. A. G.; Herbert, D. E. Deep-Red Luminescence from Platinum(II) Complexes of N^N−^N-Amido Ligands with Benzannulated N-Heterocyclic Donor Arms. *Inorg. Chem.* **2020**, *59*, 12504–12517.

(20) Ortiz, R. J.; Braun, J. D.; Williams, J. A. G.; Herbert, D. E. Brightly Luminescent Platinum Complexes of N∧C–∧N Ligands Forming Six-Membered Chelate Rings: Offsetting Deleterious Ring Size Effects Using Site-Selective Benzannulation. *Inorg. Chem.* **2021**, *60*, 16881–16894.
(21) Bruker-AXS. APEX3 V2016.1-0, 2016.
(22) Dolomanov, O. V.; Bourhis, L. J.; Gildea, R. J.; Howard, J. A. K.; Puschmann, H. OLEX2: A Complete Structure Solution, Refinement and Analysis Program. *J. Appl. Crystallogr.* **2009**, *42*, 339–341.
(23) Spek, A. L. Structure Validation in Chemical Crystallography. *Acta Cryst.* **2009**, *D65*, 148–155.
